# A Ru(ii)-arene complex with promising anti-Aβ activity

**DOI:** 10.1039/d5ra08313c

**Published:** 2026-02-03

**Authors:** Ryan M. Hacker, Jacob J. Smith, David C. Platt, Maria I. Loughlin, Emma N. Grabowski, William W. Brennessel, Marjorie A. Jones, Michael I. Webb

**Affiliations:** a Department of Chemistry & Biochemistry, SUNY Geneseo Geneseo NY 14454 USA mwebb@geneseo.edu; b Department of Chemistry, Illinois State University Normal IL 61790 USA; c Department of Chemistry, University of Rochester Rochester NY 14627 USA

## Abstract

Agents that target the amyloid-beta (Aβ) peptide associated with Alzheimer's disease have seen renewed interest following recent the clinical success of monoclonal antibody therapeutics. Metal complexes are particularly promising for development, given their relative ease of preparation and modular scaffold. Additionally, Aβ has been shown to coordinate endogenous metal ions in solution, while metal complexes can exploit this affinity, thereby modulating the aggregation of the peptide. Herein, a series of five ruthenium(ii)-arene complexes with 1,10-phenanthroline (phen) ligands were prepared and studied for their respective abilities to impact the aggregation of Aβ. Overall, the complex with the 4,7-diamino-1,10-phenanthroline ligand (RuPA) had the greatest impact on Aβ aggregation. Furthermore, this complex also displayed interactions with imidazole in aqueous media, which suggests that coordinate interactions with the peptide occur *via* histidine. Lastly, RuPA also demonstrated exceptional biocompatibility towards two neuronal cell lines and displayed a lower affinity to human serum albumin in comparison to ibuprofen. Taken together, the primary amine groups on the phen ligand enhanced the anti-Aβ ability of the complex, which is an important structure–activity relationship.

## Abstract 2

A novel ruthenium(ii)-arene complex was shown to significantly modulate the aggregation of the amyloid-beta peptide.

## Graphical abstract

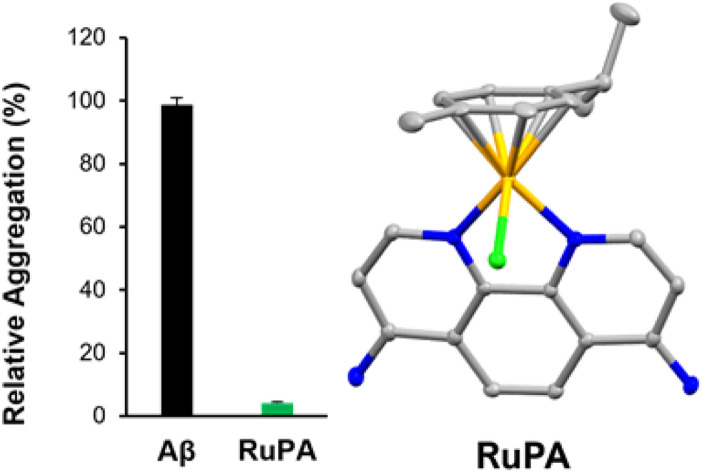

## Introduction

Currently, approximately 55 million people worldwide live with some form of dementia, of which Alzheimer's disease (AD) is the most common.^1^ The major neuropathological hallmarks of AD are neurofibrillary tangles due to abnormal tau protein accumulation and the formation of extracellular amyloid-β (Aβ) plaques on neurons.^2^ Aβ is generated following proteolytic cleavage of the amyloid precursor protein (APP) by secretases.^3,4^ These monomers are typically 40 or 42 amino acids in length, with Aβ_40_ being found in higher concentrations within diseased individuals.^5^ Both variants will spontaneously aggregate, initially forming soluble oligomers, which further assemble into protofibrils, then ultimately leading to insoluble Aβ fibrils.^6^ Indeed, it was the observation of these plaque species within the brain of an individual for which Dr Alzheimer made his seminal discovery of the disease which bears his name.^7^

Within the Aβ peptide are three histidine residues (His-6, His-13, His-14) which have been shown to coordinate endogenous metals in solution, which impact its aggregation.^8^ Metal-based therapeutics can exploit this affinity, by forming a coordinate complex with Aβ and thereby modulating its aggregation.^9^ In particular, metal complexes can form multiple interactions with Aβ, *via* metal coordination, along with secondary interactions involving the ligand(s). First-generation platinum anti-Aβ agents identified the importance of π–π interactions with the Aβ peptide,^10^ while these have also been observed with a diruthenium paddlewheel complex.^11^ By contrast, small molecule ruthenium complexes have demonstrated the importance of having primary amines on a coordinated heterocyclic ligand, resulting in improved anti-Aβ activity, which was due to hydrogen-bonding interactions with the peptide.^12–14^ While the majority of these ligands were monodentate, a study using the bidentate 2,2-bipyridine (bpy) ligand provided additional support for such interactions.^15^ Additional Ru complexes with polypyridyl ligands have shown promising anti-Aβ activity *via* copper chelation,^16^ while others are photoreactive.^17^

**Fig. 1:**
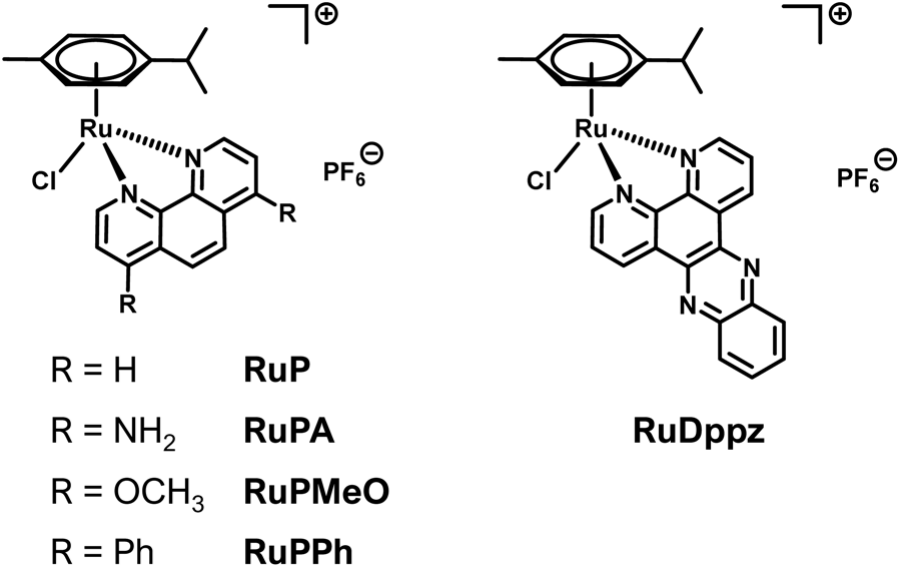
The Ru complexes prepared and evaluated herein for their respective anti-Aβ abilities.

To expand the scope of ruthenium complexes as anti-Aβ agents, a small series of “piano-stool” Ru(ii)-arene compounds were prepared and studied (Fig. 1). The ligands used were 1,10-phenanthroline (phen), where substitution at the 4′ and 7′ positions will be used to establish structure–activity relationships (SAR). Additionally, the inclusion of a dipyrido[3,2-a:2′,3′-c]phenazine (dppz) ligand will complete the series of complexes. This study will establish additional SAR between the phen complexes and their predecessor bpy analogs in determining the optimal ligand scaffold for anti-Aβ activity.

## Experimental

### Materials and methods

All materials were purchased from vendors and used directly unless specified otherwise. Chemicals used in synthesis and biological assays were obtained from Ambeed (4,7-dichloro-1,10-phenanthroline, 4,7-dimethoxy-1,10-phenanthroline, 4,7-diphenyl-1,10-phenanthroline, 1,10-phenanthroline-5,6-dione), Oakwood Chemical (1,1,1,3,3,3-hexafluoro-2-propanol (HFIP), ammonium hexafluorophosphate, warfarin), Sigma-Aldrich (ruthenium(iii) chloride hydrate, 1,10-phenanthroline, 1,2-phenylenediamine), TCI America (dansyl glycine), and Thermo Fisher (alpha-terpinene, chloroform-D, diethyl ether, dimethyl sulfoxide, dimethyl sulfoxide-D_6_, deuterium oxide, hexanes, methanol, urea). Human serum albumin was purchased as a lyophilized powder from Sigma-Aldrich. Aβ_40_ was purchased from GenScript. Elemental analysis (EA) was collected at the University of Rochester's Center for Enabling New technologies Through Catalysis using a PerkinElmer 2400 Series II Analyzer. ^1^H, ^13^C NMR (in DMSO-D_6_ or CDCl_3_), and D_2_O/DMSO-D_6_^1^H NMR spectra were obtained using a Varian 400-MR 400 MHz NMR spectrometer.

### Synthesis of the ligands

The synthesis of 4,7-diamino-1,10-phenanthroline and dipyrido[3,2-a:2′,3′-c]phenazine followed previous procedures.^18,19^

### Synthesis of the Ru complexes

The ruthenium(ii)-arene dimer ([Ru(η^6^-*p*-cymene)Cl_2_]_2_) was synthesized as previously described.^20^ A general synthesis for the Ru complexes is provided: Ru-dimer (0.1000 g, 0.163 mmol) was added to the respective derivatized phenanthroline ligand (0.326 mmol) with methanol (12.5 mL) and stirred for 1 h. Next, ammonium hexafluorophosphate (0.489 mmol) was dissolved in methanol (5 mL) and added to the solution dropwise and stirred for an additional 1 h. Storage at −20 °C overnight resulted in precipitation of the desired product which was isolated by vacuum filtration and washed with diethyl ether. The samples were dried under high vacuum for at least 6 h prior to characterization.

#### RuP

((Ru(η^6^-*p*-cymene)(1,10-phenanthroline)Cl)PF_6_) Orange zest solid (0.0946 g, 48.6% yield). ^1^H NMR (400 MHz, DMSO-D_6_, ppm): 9.89 (2H, dd), 8.90 (2H, dd), 8.26 (2H, s), 8.14 (2H, dd), 6.31 (2H, d), 6.08 (2H, d), 2.56 (1H, sept), 2.13 (3H, s), 0.87 (6H, d). ^13^C NMR (100 MHz, DMSO-D_6_, ppm): 18.62, 22.06, 30.83, 84.23, 86.31, 103.04, 104.46, 126.83, 127.91, 130.47, 139.20, 145.53, 156.34. EA results for C_22_H_22_N_2_ClRuPF_6_ theoretical: 44.34 C, 3.72 H, 4.70 N. Experimental: 44.07 C, 3.70 H, 4.63 N.

#### RuPA

Similar ratios were used in the synthesis of RuPA; however, the resulting mixture was heated to reflux for 16 hours, then cooled to room temperature and immediately filtered to isolate the desired product. Crystals suitable for X-ray diffraction were isolated from the reaction filtrate following prolonged storage at −20 °C. ((Ru(η^6^-*p*-cymene)(4,7-diamino-1,10-phenanthroline)Cl)PF_6_) Marigold-yellow powder (0.1033 g, 98.7% yield). ^1^H NMR (400 MHz, DMSO-D_6_, ppm): 8.98 (2H, d), 8.07 (2H, s), 7.81 (4H, s), 6.89 (2H, d), 5.98 (2H, d), 5.75 (2H, d), 2.10 (3H, s), 0.85 (6H, d). ^13^C NMR (100 MHz, DMSO-D_6_, ppm): 18.74, 22.08, 30.73, 82.66, 85.11, 101.34, 101.52, 107.31, 118.60, 119.01, 146.67, 153.72, 154.15. EA results for C_22_H_24_N_4_ClRuPF_6_·0.35CO(NH_2_)_2_ theoretical: 41.49 C, 3.96 H, 10.18 N. Experimental: 41.34 C, 4.31 H, 9.91 N.

#### RuPMeO

Crystals suitable for X-ray diffraction were isolated from the reaction filtrate following prolonged storage at −20 °C. ((Ru(η^6^-*p*-cymene)(4,7-dimethoxy-1,10-phenanthroline)Cl)PF_6_) Curry red powder solid (0.1391 g, 63.7% yield). ^1^H NMR (400 MHz, DMSO-D_6_, ppm): 9.64 (2H, d), 8.20 (2H, s), 7.59 (2H, d), 6.21 (2H, d), 5.89 (2H, d), 4.24 (6H, s), 2.55 (1H, sept), 2.14 (3H, s), 0.88 (6H, d). ^13^C NMR (100 MHz, DMSO-D_6_, ppm): 18.70, 22.11, 30.82, 58.26, 83.25, 85.65, 102.41, 103.16, 107.64, 120.69, 122.12, 146.10, 157.54, 163.94. EA results for C_24_H_26_N_2_O_2_ClRuPF_6_ theoretical: 43.94 C, 4.00 H, 4.27 N. Experimental: 43.51 C, 3.89 H, 4.16 N.

#### RuPPh

((Ru(η^6^-*p*-cymene)(4,7-diphenyl-1,10-phenanthroline)Cl)PF_6_) Cadmium yellow solid (0.1931 g, 77.9% yield). ^1^H NMR (400 MHz, CDCl_3_, ppm): 9.64 (2H, d), 8.03 (2H, s), 7.94 (2H, d), 7.57 (10H, m), 6.06 (2H, d), 5.88 (2H, d), 2.83 (1H, sept), 2.24 (3H, s), 1.15 (6H, d). ^13^C NMR (100 MHz, CDCl_3_, ppm): 18.62, 22.05, 31.26, 84.96, 86.19, 101.71, 106.08, 110.00, 125.66, 126.71, 128.72, 129.22, 129.74, 130.04, 134.94, 146.40, 151.40, 154.64. EA results for C_34_H_30_N_2_ClRuPF_6_ theoretical: 52.08 C, 4.37 H, 3.57 N. Experimental: 52.23 C, 4.08 H, 3.73 N.

#### RuDppz

((Ru(η^6^-*p*-cymene)(dipyrido[3,2-a:2′,3′-c]phenazine)Cl)PF_6_) Dijon mustard yellow solid (0.0900 g, 76.8% yield). ^1^H NMR (400 MHz, DMSO-D_6_, ppm): 9.98 (2H, q), 9.70 (2H, dd), 8.45 (2H, dd), 8.28 (2H, dd), 8.13 (2H, dd), 6.36 (2H, d), 6.13 (2H, d), 2.66 (1H, sept), 2.18 (3H, s), 0.96 (6H, d). ^13^C NMR (100 MHz, DMSO-D_6_, ppm): 18.69, 22.21, 30.88, 84.65, 86.38, 103.22, 105.22, 128.08, 129.88, 129.94, 133.02, 135.80, 139.78, 142.43, 148.46, 157.76. EA results for C_28_H_24_N_4_ClRuPF_6_ theoretical: 45.82 C, 3.84 H, 7.63 N. Experimental: 46.10 C, 3.55 H, 7.65 N.

### Log *D*

A stock solution was prepared for each Ru complex using dimethyl sulfoxide (DMSO) and diluted to 6 mL at a concentration of 50 µM using phosphate buffered saline (PBS, pH 7.4), with the final DMSO concentration being less than 5%. The absorbance spectrum was measured, then 6 mL of 1-octanol was added, and the resulting biphasic sample was mixed at room temperature for 2 hours using an IKA Trayster inversion mixer (60 rpm). Next, the aqueous layer was extracted, and its absorbance spectra was measured. Log *D* values were determined using the equation below with samples prepared and analyzed in triplicate:
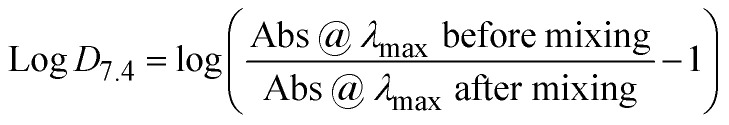


### UV-vis sample preparation

A stock solution was prepared for each Ru complex using DMSO and PBS, then diluted with PBS to 100 µM, where the final concentration of DMSO was less than 5%. UV-vis spectra were collected using a Cary 50 spectrophotometer with measurements from 220–800 nm. For the duration of the experiment, samples were capped and incubated at 37 °C using an attached single cell Peltier system to maintain temperature. The absorbance of each sample was measured at 1 scan every minute for 30 minutes, followed by 1 scan every 10 minutes for up to 6 hours.

### Thioflavin T fluorescence assay

The Aβ aggregation assay was performed as previously described.^25,28^ All of the PBS used was filtered through a 0.20 µm Titan 3 syringe filter prior to its use. Ru stock solutions were prepared by dissolving approximately 0.0015 g of each complex in 0.50 mL DMSO and diluting to 10 mL with PBS. Thioflavin T (ThT) was prepared in a similar manner but was protected from the light and additionally filtered prior to the assay. Aβ was monomerized following previous procedures^21^ and stored at −20 °C until use. To prepare a stock solution of Aβ, this was first removed from the freezer and placed on finely crushed ice for 15 minutes. The sample was taken off ice and 15 µL DMSO was added and gently flicked to allow the DMSO to dissolve the Aβ residue. PBS was then added to the sample and sonicated at room temperature for 20 minutes, or until the solution was clear. Using an Implen NanoPhotometer N50 at *λ* = 280 nm, the concentration of the Aβ stock was determined using the extinction coefficient of 1490 M^−1^ cm^−1^.^22^ Solutions were prepared on a black clear-bottom 96-well plate with each well having a final volume of 150 µL. Aliquots from stock solutions were plated into the wells so that the concentrations of Ru, ThT, and Aβ were all 10 µM. The order in which solutions were added to the plate was PBS, Ru complexes, ThT, and Aβ. ThT fluorescence was measured using a SpectraMax M3 plate reader with *λ*_ex_ = 440 nm and *λ*_em_ = 480 nm, where the temperature was maintained at 37 °C. Measurements were taken every 30 minutes for a total of 8 hours. Statistical analysis was conducted using a one-way analysis of variance (ANOVA).

### DLS sample preparation

At the conclusion of the Aβ aggregation assay, samples were taken directly from the 96-well plate and measured by dynamic light scattering (DLS). For this, a 125 µL aliquot extracted from a desired well was placed into a 1 mL syringe and passed through a syringe filter (pore size: 0.2 µm) into a 0.5 mL microcentrifuge tube. The sample was then pipetted into a polystyrene cuvette and DLS spectra were measured using a Malvern Zetasizer Nano ZSP. Measurements were represented as the average of percent intensity through several runs that were determined by the Zetasizer software Version 7.13.1.

### TEM sample preparation

Following DLS data collection, these samples were used to prepare grids for imaging by transmission electron microscopy (TEM). An aliquot (10 µL) from a DLS sample was deposited onto a formvar-coated copper grid and allowed to settle for 120 seconds. The solvent was wicked away, and the sample was stained using a 2% uranyl acetate solution (10 µL). This was wicked away after 60 seconds and the grid was washed with one aliquot of water (10 µL) which was left for 30 seconds before being wicked away. The grids were then stored at room temperature, before being measured using a high-resolution JEOL JEM 2100F operating at 200 kV.

### Protein binding assay

Fluorescent quenching experiments were conducted using a PTI QuantiMaster 50 by measuring the displacement of a fluorescent probe bound to human serum albumin (HSA). Sudlow sites I and II were analyzed using the fluorescent probes warfarin (WF) and dansyl glycine (DG). Stock solutions of both probes were prepared separately where approximately 1 mg of each respective probe was initially dissolved in DMSO and diluted with PBS to have a final DMSO concentration of <1%. HSA was prepared by dissolving approximately 40 mg of lyophilized HSA powder in 6 mL of PBS and gently mixed using an IKA Trayster inversion mixer (60 rpm) until dissolved. Stock solutions of each Ru complex were prepared by dissolving 1.5 mg in DMSO and diluting with PBS resulting in <5% DMSO. Each sample had 2.5 µM HSA and 2.5 µM WF or DG while maintaining a volume of 3500 µL. Aliquots of the respective Ru complexes were added to samples where the concentration increased from 0 to 62.5 µM in 12.5 µM increments. Samples were prepared through the sequential addition of PBS, HSA, DG/WF, and the Ru complex with the final concentration of DMSO being <2%. Then, samples were mixed by the inversion mixer for 30 seconds prior to incubation in the Neslab gp-200 for 15 minutes at 28 °C and then mixed again for 30 seconds on the inversion mixer. Samples were then measured at room temperature where the excitation wavelength was 295 nm for WF, with an emission range of 310–500 nm, a step of 1 nm, and an integration time of 0.2 seconds. The parameters for DG maintained the same step and integration time, but had an excitation wavelength of 335 nm and an emission spectrum of 350–600 nm.

### Cytotoxicity testing

Cell viability screening was done using a standard MTT (3-(4,5-dimethylthiazol-2-yl)-2,5-diphenyltetrazolium bromide) assay using adherent *Rattus norvegicus* pheochromocytoma cells (PC-12; ATCC CRL-1721) and axenic *Rattus norvegicus* C6 glioma cells (ATTC CCL-107) following previous procedures.^15^

## Results and discussion

### Synthesis and characterization

**Fig. 2:**
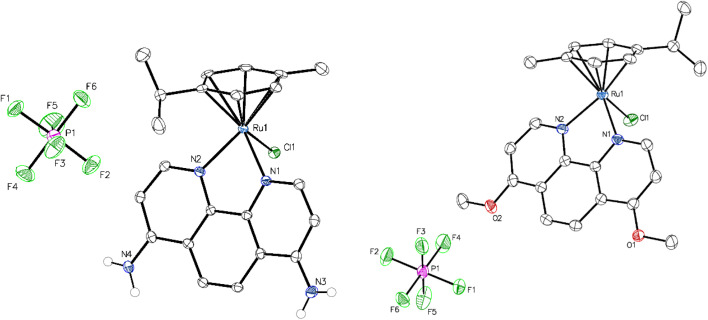
X-ray crystal structures of RuPA (left) and RuPMeO (right). The ellipsoids of all non-hydrogen atoms are shown at the 50% probability level.

All of the Ru complexes were prepared by the mixing of a Ru(ii)-arene dimer with the respective phenanthroline derivatives, resulting in five compounds, of which RuPA is novel, while RuPMeO has been minimally characterized previously.^23^ Preliminary characterization of all the complexes was achieved using ^1^H and ^13^C NMR spectroscopy (Fig. S1–S10). For RuP, RuPPh, and RuDppz, comparisons were made to previous reports,^24,25^ which confirmed their successful synthesis, while elemental analysis established bulk purity. Furthermore, crystals suitable for X-ray crystallography were isolated for both RuPA and RuPMeO (Fig. 2). Both structures had the expected “piano-stool” configuration around the ruthenium metal center. For this, one side of the metal center is occupied by the η^6^-arene ring, while the other has the bidentate phen ligand with coordination *via* the nitrogen atoms, and a single chloride ligand to complete the coordination sphere. The metal ligand bond lengths for the coordinated phen ligands are similar between the two complexes, with RuPA having a Ru–N bond length of 2.0944 Å while for RuPMeO the average Ru–N bond length is 2.0986 Å. By contrast, the Ru–Cl bond length has a pronounced difference between the two complexes, with RuPA having a longer bond of 2.4410(6) Å, compared to that of RuPMeO with 2.3913(6) Å. This difference is likely due to the differences in the electron donating groups, methoxy and primary amine, which result in a longer Ru–Cl bond length for RuPA. Upon comparing the Ru–N and Ru–Cl bond lengths to the previously solved crystal structures of RuP, RuPPh, and RuDppz,^24,25^ similar Ru–N bond lengths were observed to that of RuPMeO, while the Ru–Cl bond of RuPA is the longest for this series of compounds.

### Aqueous stability and partitioning

Metallotherapeuics are often categorized as prodrugs, whereby activation of the complex is achieved *in situ via* ligand exchange.^26^ Indeed, for ruthenium therapeutics such as previous anticancer complexes that have seen clinical success,^27^ aqueous ligand exchange of a chloride often precedes coordination to the ultimate biological target.^28^ To evaluate the relative stability of the prepared Ru complexes two complementary spectroscopic methods were used: ^1^H NMR and UV-vis absorption. Beginning with the UV-vis measurements, 100 µM samples of each complex were prepared in PBS and incubated at 37 °C for up to 6 hours. For all five complexes, no substantial changes were observed within the UV-vis spectra for the duration of the experiments (SI Fig. S11–S15). For the NMR studies, a substantially higher concentration of each complex was required (∼18 mM); however, this necessitated the use of elevated amounts of DMSO to ensure solubility. This strongly correlated with the hydrophobicity of the complexes, as RuP, RuPA, and RuPMeO used 33% DMSO, RuDppz needed 50% DMSO, while for RuPPh 75% DMSO was necessary to maintain aqueous solubility. DMSO is unique, in that it is polar, aprotic, and miscible with water, while also being able to dissolve a wide variety of polar and non-polar molecules.^29^ Following initial dissolution in DMSO and dilution using D_2_O, the ^1^H NMR spectra for each respective complex were measured following 0, 1, 6, and 24 hours of incubation at 37 °C. Initially, for most of the complexes, no substantial ligand exchange was observed; however, with extended incubation new signals emerged within the spectrum in close proximity to those of the parent molecule. These are indicative of aquation and are assigned to the exchange of the single chloride ligand for a water molecule, as the signals from the bound arene ring and phen ligands remain prominent and the new signals do not match those of the respective free ligands, indicating that no dissociation occurred. Indeed, a previous study which included both RuP and RuPPh evaluated the aqueous ligand exchange of the complexes, where it was found that the chloride ligand is replaced with a water molecule.^30^ For both complexes, neither the arene, nor the phen ligand, were replaced following prolonged aqueous incubation, which are in agreement with our results.

Overall, the relative extent and rate of exchange was consistent for most of the complexes, except for RuPA, where rapid exchange was immediately observed following dissolution in aqueous media. One explanation for this is within the crystal structure (Fig. 2), where a relatively long Ru–Cl bond was observed, due to the electron donating primary amine groups, which are ultimately activating the metal center for aqueous exchange. Similar behavior was previously reported for RuP and RuPPh, where the aqueous half-life of RuPPh was substantially less than that of RuP.^30^

A common pharmacological metric for drugs is their ability to partition between hydrophobic and hydrophilic environments.^31^ This phenomenon can be quantified using the partitioning coefficient, log *P*, which is measured *via* the mixing of a molecule in a biphasic mixture of 1-octanol and water. For ruthenium complexes, pH can play an important role in the stability and speciation of the complexes,^32,33^ therefore a log *D* was performed. Furthermore, ligand exchange around the metal center can be rather rapid (*vide supra*), therefore mixing of the complexes for the partitioning was performed for only 2 hours. This was in an effort to minimize the extent of aqueous ligand, thereby primarily capturing the partitioning of the parent molecule. This has been shown to be an effective method to measure partitioning of ruthenium complexes.^34^ Furthermore, for these studies buffered aqueous media (PBS) was utilized, as this would aide in forestalling aqueous exchange *via* the chloride ions within the media, while also providing a more biologically-relevant picture of the partitioning.

Overall, for the prepared complexes the measured partitioning followed the anticipated trend where the more polar functional groups on the phen ligand gave hydrophilic complexes, while the non-polar substituents yielded hydrophobic complexes. These values are summarized in Table 1. The lone outlier was the measured log *D* for RuP, as this was more hydrophilic than RuPA, despite the presence of the polar primary amines on RuPA. In order to achieve passive diffusion across the blood–brain barrier (BBB), a partitioning of 1–3 has been codified as being optimal.^35^ Two of the prepared complexes, RuPPh and RuDppz, are within this range, while RuPMeO is just outside of it. This is an encouraging result, and the values reported are substantially more lipophilic than 2,2-bipyridine analogs,^15^ highlighting the impact of the conjugated phen ligand backbone. Furthermore, the presence of a positive charge at pH 7–8 has been shown to have a positive correlation with BBB passage,^36^ a property which all of the prepared complexes possess as complex ions.

**Table 1:** Partitioning values (log *D*_7.4_) and protein binding constants (log *K*′) for the prepared complexes

	Log *D*_7.4_	log *K*′ (DG)	log *K*′ (WF)
RuP	−0.78 ± 0.04	3.86	4.26
RuPA	−0.06 ± 0.06	4.01	4.39
RuPMeO	0.36 ± 0.01	3.92	4.00
RuPPh	1.76 ± 0.21	3.98	4.39
RuDppz	1.03 ± 0.09	4.45	5.20

The full-length Aβ peptide contains three histidine residues (His-6, His-13, and His-14) which have been shown to coordinate endogenous metal ions and previous therapeutic metal complexes.^37,38^ Indeed, it is anticipated that these histidine residues are the likely site of coordination for the prepared Ru complexes, given that previous metal-based complexes which target Aβ have been shown to bind at these sites, a phenomenon which is accredited to forestalling the aggregation of the peptide.^39^ To evaluate the ability of the complexes to target the histidine residues of Aβ, ^1^H NMR studies were performed, where the complexes were mixed with an equimolar amount of imidazole, as this is the functional group for histidine. The sample preparation mirrored that of the solution studies, as DMSO was also used to ensure solubility was maintained. This facilitated the comparison between the ^1^H NMR spectra in the presence and absence of imidazole.

Following initial dissolution and mixing, no changes were observed within the ^1^H NMR spectrum for RuP (Fig. S21), RuPPh (Fig. S24), or RuDppz (Fig. S25), while for RuPMeO (Fig. S23) new signals began to emerge within the spectrum. After one hour, new peaks were observed for RuP and RuDppz, although these were still minor features. For RuPPh no new signals were observed, which is accredited to the elevated amount of DMSO used for this experiment (75%), which is identical to that of the D_2_O study where the spectra were devoid of change. By contrast, for RuPMeO, the new signals initially observed rose to become equivalent in intensity to those of the parent complex.

**Fig. 3:**
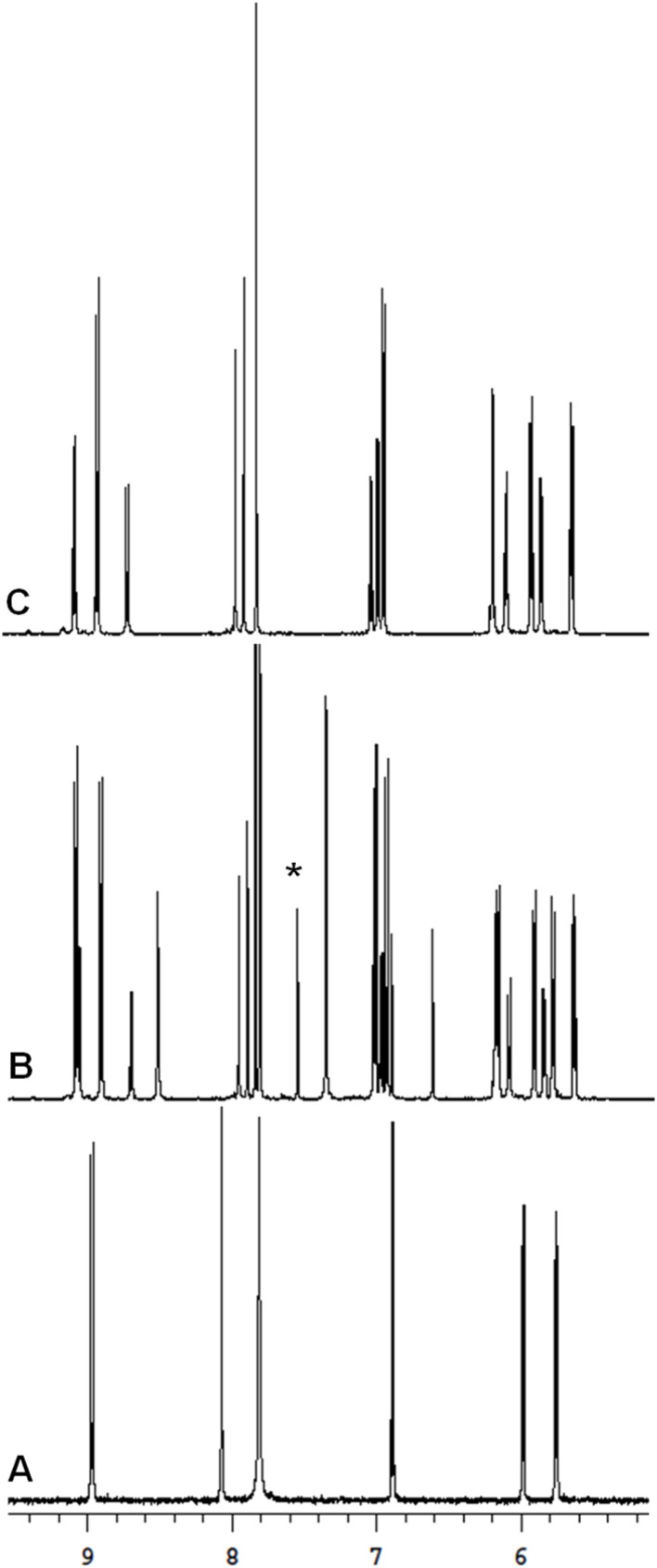
^1^H NMR spectra of the aromatic region for RuPA where: (A) RuPA in DMSO-D_6_, (B) RuPA (18 mM) with imidazole (18 mM) with no incubation at 37 °C, and C. RuPA in D_2_O with no incubation. Free imidazole peaks are marked with an asterisk (*).

Taken together, the spectra for the above four complexes are in stark contrast to that of RuPA (Fig. 3), where new signals were prominently observed within the spectrum even in the absence of incubation. These signals were in proximity to those from the parent complex and of similar intensity. After one hour of incubation, these new signals effectively replaced those of the parent complex, becoming the predominant features observed within the spectrum (Fig. S22). Such signals were not observed in D_2_O alone, and are assigned to an imidazole-coordinated complex, which formed *via* the replacement of the chloride ligand. This is an encouraging result, as the complexes are anticipated to coordinate to Aβ *via* histidine, which should thereby result in a modulation of its aggregation.

### ThT fluorescence

**Fig. 4:**
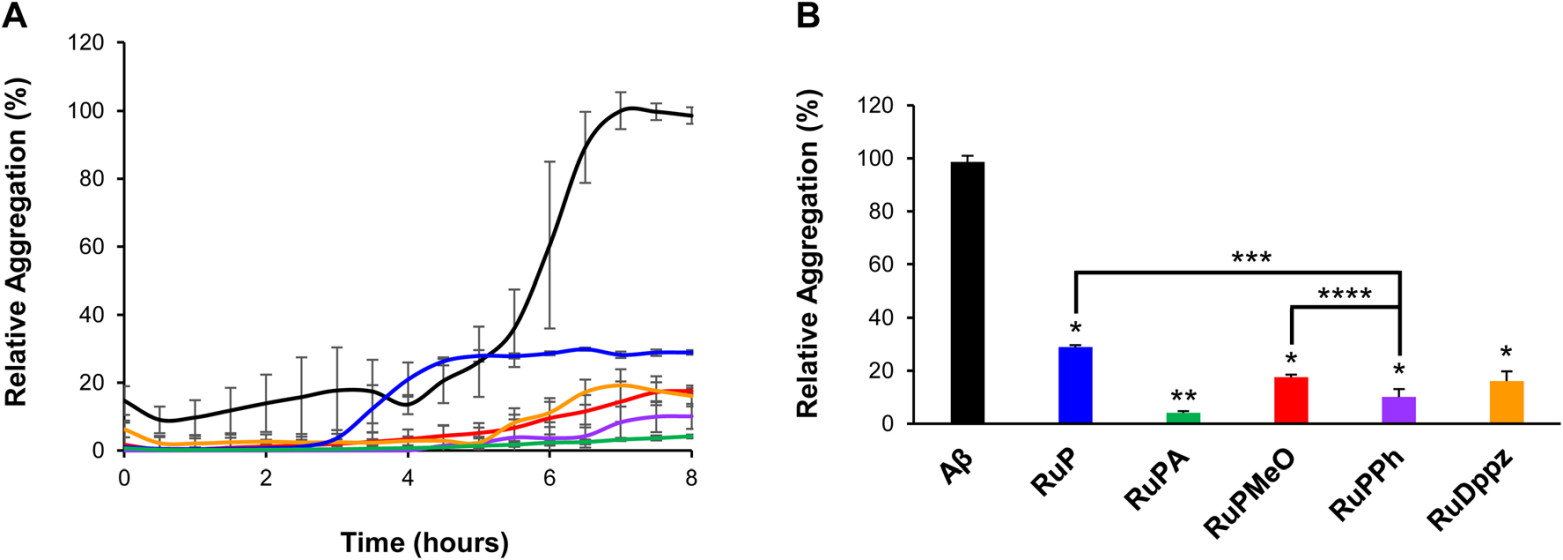
ThT fluorescence results following the incubation of equimolar solutions (10 µM) of Aβ_40_ with the Ru complexes for up to 8 h at 37 °C. Where (A) is the full data set, and (B) is a plot of the 8 h time point. The signals were normalized to the positive control of the free peptide in solution at the 8 h time point. The error bars represent the standard deviation observed for each data point for the respective samples. Each sample was measured in triplicate, where black = Aβ_40_ alone; blue = Aβ_40_ + RuP; green = Aβ_40_ + RuPA; red = Aβ_40_ + RuPMeO; purple = Aβ_40_ + RuPPh; orange = Aβ_40_ + RuDppz. All statistical analysis was performed using a one-way ANOVA, where *P < 0.05 for the treatments relative to Aβ40 alone, **P < 0.001 for RuPA relative to the other Ru complexes, ***P < 0.05 for RuPPh relative to RuP, and ****P < 0.05 for RuPPh relative to RuPMeO.

To determine the potential of each complex to modulate the aggregation of Aβ_40_ an aggregation assay was performed. For this, each complex was mixed with an equimolar ratio of Aβ_40_ along with the fluorometric probe thioflavin T (ThT). In the presence of peptide aggregates, ThT emits a characteristic fluorescence signal, which has been noted to correlate with Aβ aggregation.^40^ For the assay, samples were prepared where an equimolar amount of the respective Ru complex was mixed with Aβ_40_ in PBS, and incubated at 37 °C, with fluorescence measurements taken every 30 minutes for up to 8 hours. In the absence of any Ru complex, Aβ_40_ alone displayed the anticipated sigmoidal curve, whereby the fluorescence intensity spiked after 6 hours of incubation, then plateaued towards 8 hours (Fig. 4A). Comparatively, with the majority of the respective Ru complexes no substantial increase in the fluorescence intensity was observed. While an overall increase in fluorescence did occur around 6 hours and plateau by the 8 hour mark, this change was significantly diminished compared to that of Aβ_40_ alone. This is highlighted in Fig. 4B, where the signals after 8 hours of incubation are plotted against each other. From these signals, a ranking of the activity of the complexes was established, based upon the relative percent aggregation of Aβ, where RuP (28.9% ± 0.7%) < RuPMeO (17.6% ± 0.8%) < RuDppz (16.1% ± 3.7%) < RuPPh (10.1% ± 3.0%) < RuPA (4.2% ± 0.5%). Compared to previous ruthenium-based anti-Aβ complexes,^41^ the performance of RuPA ranks among the best prepared to date.

### Particle size analysis

**Fig. 5:**
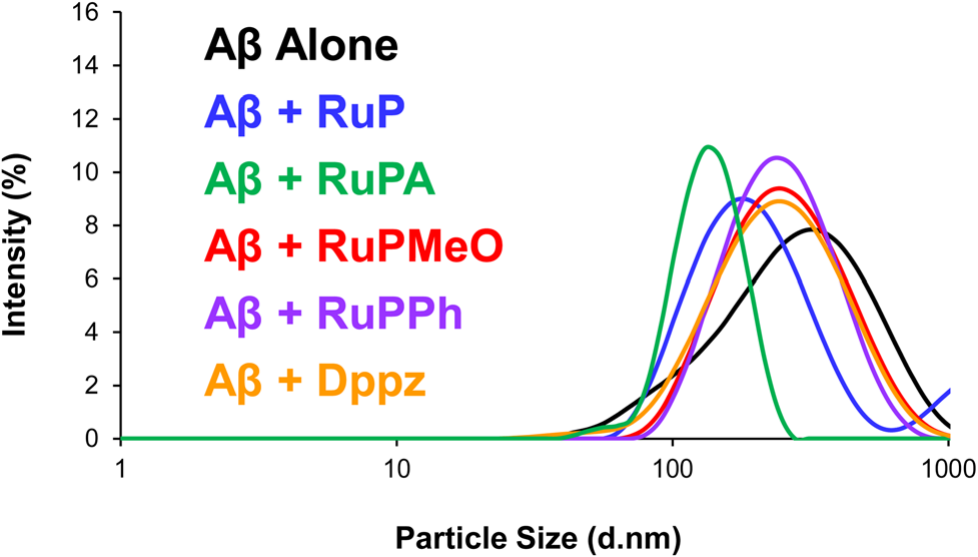
Particle size distribution (hydrodynamic radii, *d* nm) of Aβ_40_ in the absence and presence of the respective Ru complexes for 8 hours at 37 °C in PBS, where black = Aβ_40_ alone, blue = Aβ_40_ + RuP, green = Aβ_40_ + RuPA, red = Aβ_40_ + RuPMeO, purple = Aβ_40_ + RuPPh, orange = Aβ_40_ + RuDppz.

The aggregation of Aβ can lead to a variety of particle sizes in solution,^42^ which can be quantified using dynamic light scattering (DLS), where the Brownian motion of the particles following exposure to laser light results in a size distribution profile. Additionally, the polydispersity (PDI) obtained from a DLS measurement is often used as a parameter for predicting protein crystal growth formation,^43^ making it amendable to monitor protein aggregation.^44^ Indeed, DLS has been previously used to determine the size of the Aβ monomer (1–4 nm),^45^ while subsequent aggregates species are substantially larger in size.^46^ Following the completion of the ThT fluorescence assay, samples were filtered through a 0.2 µm filter and measured using DLS, whereby differences following the treatment with the respective Ru complexes will provide insight into their ability to modulate the aggregation of the peptide. In the absence of any Ru complex, Aβ_40_ had a single peak at 327 nm (PDI = 0.271), while in the presence of the Ru complexes a discernible shift to smaller particle sizes was consistently observed (Fig. 5). This was particularly pronounced for RuPA, which had a prominent peak separate from most of the Ru complexes corresponding to an average particle size of 139 nm (PDI = 0.615). By contrast, the remaining four complexes had particles sizes predominantly above 200 nm, three of which were virtually superimposed on one another, with RuPPh at 268 nm (PDI = 0.196), RuDppz at 274 nm (PDI = 0.243), and RuPMeO at 285 nm (PDI = 0.222)Lastly, RuP had particle sizes of 209 nm (PDI = 0.419). Unsurprisingly, in all cases, the PDI values indicated that the samples were polydisperse, with varied sizes and potentially aggregated species. These results reflect those of the ThT assay, whereby RuPA again yielded the greatest effect on Aβ_40_ aggregation. Using the measured particle sizes, a ranking of the complexes for their ability to modulate Aβ_40_ aggregation is as following: RuPMeO (285 nm) < RuDppz (274 nm) < RuPPh (268 nm) < RuP (209 nm) < RuPA (139 nm).

### Electron microscopy imaging

**Fig. 6:**
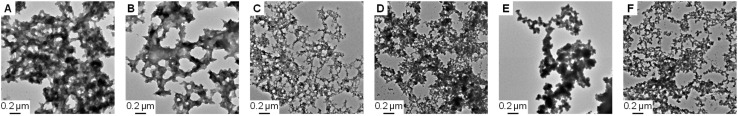
TEM images taken at 40 kX magnification of the DLS filtrates: (A) Aβ_40_ alone, (B) Aβ_40_ + RuP, (C) Aβ_40_ + RuPA, (D) Aβ_40_ + RuPOMe, (E) Aβ_40_ + RuPPh, (F) Aβ_40_ + RuDppz.

Following DLS measurements, the same samples were used to prepare grids for analysis by transmission electron microscopy (TEM). By measuring the same sample by three sequential methods, a wholistic picture of the relative impact of each Ru complex on the aggregation of the peptide is obtained. By itself, the Aβ_40_ peptide displayed large, densely packed amorphous aggregates (Fig. 6 and S26). Following treatment with the respective Ru complexes, these features were greatly diminished and dispersed, as elongated and less dense amorphous aggregates were observed. This allowed for a qualitative ranking of the complexes for their abilities to modulate the aggregation of Aβ_40_. Specifically, RuPA stood out from the pack, as the features observed following treatment with this complex were the most dispersed and least dense of all the complexes. This correlates with the DLS data, as the smallest particles were yet again observed for RuPA. For the remaining complexes, a similar disruption to the aggregate species was observed, where the relative density of the particulates and their distribution allowed for them to be ranked in terms of activity, where RuPMeO < RuP < RuPPh < RuDppz < RuPA.

### Protein binding

**Fig. 7:**
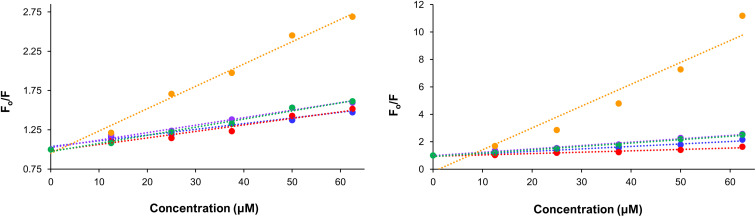
Stern–Volmer plots for the complexes after mixing them with HSA and DG (left) and WF (right). Blue = RuP, green = RuPA, red = RuPMeO, purple = RuPPh, orange = RuDppz.

Human serum albumin (HSA) is one of the most abundant proteins within human plasma, and an important target for several metallotherapetics.^47^ However, for Alzheimer's therapeutics this is antithesis to their activity, as HSA does not readily cross the BBB.^48^ Therefore, the associations of a potential neurotherapeutic with this protein should be minimized in order to promote BBB passage. The affinity of a compound for HSA can be determined using a fluorescence displacement assay, whereby two common binding sites on HSA are studied. The first binding site, commonly referred to as Sudlow Site I,^49^ prefers bulky aromatic molecules like warfarin (WF) and Sudlow site II,^50^ which prefers flat aromatic molecules like dansyl glycine (DG). The displacement of either probe from their preferred binding site *via* the sequential addition of increasing amounts of the respective Ru complexes (Fig. S27–S36) was used to construct a Stern–Volmer plot (Fig. 7), from which a conditional binding constant for that particular site on HSA was obtained. The results are summarized in Table 1, where the binding constants (log *K*′) ranged from 3.86 to 5.20.

In general, for all five complexes, greater affinity for Sudlow site I was observed, as anticipated based upon the bulky nature of the phen ligands. While for the individual complexes, the weakest and strongest affinity mirrored the phen ligands and log *D*_7.4_ values, where the lowest binding affinity for HSA at both binding sites was observed for RuP, while the greatest binding affinity was observed for RuDppz. For the remaining complexes, remarkably similar binding constants were measured for Sudlow Site II, while for Sudlow Site I RuPMeO had the lowest affinity, while RuPA and RuPPh had identical values. This is in stark contrast to the hydrophobicity of the complexes, and an interesting result for RuPA, as the polar primary amine groups were expected to minimize interactions with HSA, specifically within the targeted hydrophobic pockets. Taken together, the measured binding constants for the Ru complexes were lower affinity for HSA when compared with ibuprofen, a pain relief drug which is known to permeate the BBB, which has a binding constant of 5.23 and 6.30 for Sudlow Sites I and II respectively.^51^

### Neuronal cell toxicity

A final evaluative measure in the performance of the complexes was to determine their tolerance by two neuronal cell lines using a standard MTT cell viability assay. The first was a glial cell line, as these are the most abundant cell type within the brain,^52^ while the second was PC12 chromaffin cells, which are sensitive to oxidative stress.^53^ These cell lines have been previously used in the evaluation of other Ru anti-Aβ agents,^14,15,41,54^ making them a suitable for establishing neurotoxicity. The respective cell lines were incubated with increasing amounts of the individual Ru complexes for 24 hours, following which viability measurements were taken.

**Fig. 8:**
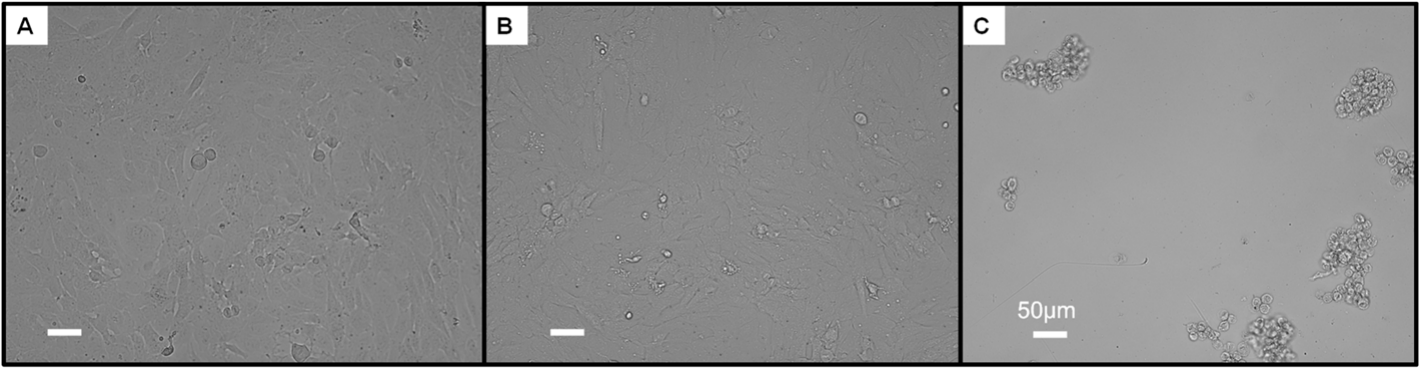
PC12 cells incubated for 24 hours with (A) DMSO control cells, (B) 40 µM RuPA, (C) 40 µM complex RuPPh.

For the C6 glioma cells, variable levels of toxicity was observed across the complexes, which was often dose-dependent (Fig. S37). For these cells, RuPA had the highest viability with 89.9 ± 7.4% at 40 µM, followed by RuP (57.1 ± 4.0%), RuPMeO (45.5 ± 2.4%), RuDppz (21.0 ± 3.6%), and RuPPh (1.1 ± 0.3%). Similarly, towards the PC12 adrenal cells the complexes again displayed dose-dependent toxicity (Fig. S38); however, the average viability was higher than the C6 cells. At the highest dose administered (40 µM), the order of the complexes by increasing cell viability was: RuPPh (1.7 ± 1.0%), RuDppz (18.5 ± 1.1%), RuPMeO (78.5 ± 6.1%), RuP (84.1 ± 3.1%), and RuPA (89.8 ± 4.7%). This stark contrast in viability, particularly between RuPA and RuPPh is demonstrated within the light microscopy images, where the cells exposed to both complexes are shown in Fig. 8. Similar comparison can be made across the remaining complexes where the cell morphologies are substantially different in the presence of RuPPh in comparison to the other Ru complexes (Fig. S39–S48). Previous studies of similar ruthenium complexes containing the same 4,7-diphenyl-1,10-phenanthroline or dppz ligands also saw substantial cytotoxicity towards cancer cell lines.^25,55^ Both such ligands promote interactions with DNA, in particular the dppz ligand which facilitates extensive intercalative stacking with DNA base pairs.^56^ Taken together, using the measured cells viabilities towards both cell lines, the biocompatibility of the Ru complexes can be ranked, where: RuPA > RuP > RuPMeO > RuDppz > RuPPh.

## Conclusion

The relative abilities of five Ru(ii)-arene complexes, one of which is novel, with differing 1,10-phenanthroline ligands to modulate the aggregation of the Aβ peptide was performed. Most of the complexes displayed minimal ligand exchange following dissolution and prolonged incubation within aqueous media. Similarly, upon mixing with imidazole, new signals gradually emerged within the ^1^H NMR spectrum for the majority of the complexes, indicating that coordination had occurred. This is an important consideration, as the likely interaction with the Aβ peptide occurs *via* coordination with histidine. The lone exception was RuPA, where rapid and substantial ligand exchange occurred within aqueous media. Similarly, upon mixing with imidazole new signals were immediately observed within the ^1^H NMR spectrum which differed from the parent complex alone in aqueous media, signifying that coordination had occurred following exchange of the chloride ligand. The sheer extent and speed with which this occurred for RuPA was in stark contrast to the other complexes, likely due to the primary amine groups on the phen ligand which labilize the Ru–Cl bond.

Upon mixing with Aβ_40_, all of the complexes were successful in modulating the aggregation of the peptide, resulting in less dense amorphous aggregates of smaller sizes compared to the peptide alone. Furthermore, the protein binding of the complexes was measured, where affinities similar to previous Ru(ii)-arene complexes were observed.^15^ Lastly, cytotoxicity studies identified RuP, RuPA, and RuPMeO as being well tolerated in neuronal cells, while RuPPh and RuDppz unfortunately exhibited substantial toxicity at the doses tested.

Taken together, the performance of each respective complex was ranked on a scale of 1–5 where 1 is the best and 5 is the worst. This allowed for a wholistic view of the anti-Aβ activity of the complexes, which would in turn be used to establish SAR. For these rankings, modulation of Aβ_40_ aggregation was quantified using the results from the ThT fluorescence, DLS, and TEM studies. For the log *D*_7.4_ values, a more hydrophobic complex was preferred, as which would promote passive diffusion across the BBB. Since a log *D* of 1–3 has been previously identified as desirable for a neurotherapeutic,^35^ complexes within this range received the greatest score. Those outside of this range, yet still hydrophobic, were next, while hydrophilic complexes received the lowest score. Lastly, for cytotoxicity the greatest cell viability was favored. The cumulative rankings of the complexes are shown in Table 2, where each category was given equal weight such that a wholistic view of the complexes' activity would be obtained. Taken together, RuPA emerged as the complex with the best activity overall. This agrees with previous SAR, as the primary amine groups have been shown to substantially improve the activity in comparison to other functional groups.^34^ Furthermore, the anti-Aβ activity of RuPA is remarkably similar to its 2,2-bipyridine analog RuBA;^15^ however, with the inclusion of the conjugated phenanthroline ligand RuPA is amphipathic, while RuBA is hydrophilic. This is an important result, and one that will be incorporated into further agents that target the Aβ peptide.

**Table 2:** Summary of the rankings of the prepared Ru complexes for their abilities to modulate the aggregation of Aβ_40_ (ThT fluorescence, DLS, TEM), partition between hydrophobic and hydrophilic media (Log *D*), bind to HSA, and their cytotoxicity towards C6 and PC12 cell lines, on a scale of 1 to 5, where 1 = best, and 5 = worst

Complex	ThT	DLS	TEM	Log *D*_7.4_	Cytotoxicity	Average
RuP	5	2	4	5	2	3.6
RuPA	1	1	1	5	1	1.8
RuPMeO	3	5	5	3	3	3.8
RuPPh	2	3	3	1	5	2.8
RuDppz	3	4	2	1	4	2.8

## Conflicts of interest

There are no conflicts to declare.

## Supplementary Material

RA-016-D5RA08313C-s001

RA-016-D5RA08313C-s002

## Data Availability

CCDC 2489851 and 2449928 contain the supplementary crystallographic data for this paper.^57*a*,*b*^ The data supporting this article have been included as part of the supplementary information (SI). Supplementary information is available. See DOI: https://doi.org/10.1039/d5ra08313c.
