# Supplementary material for: A Ru(ii)-arene complex with promising anti-Aβ activity
Source: RSC Adv. 2026 Feb 3;16(8):7056–65. doi: 10.1039/d5ra08313c (PMC12865786; doi:10.1039/d5ra08313c)
Supplement: RA-016-D5RA08313C-s001 [file RA-016-D5RA08313C-s001.pdf]

Supplementary Information (SI) for RSC Advances.
This journal is © The Royal Society of Chemistry 2026

Supplementary Information For:

A Ru(II)-arene Complex With Promising Anti-Aβ Activity

Ryan M. Hacker,[1] Jacob J. Smith,[1] David C. Platt,[2] Maria I. Loughlin,[1] Emma N. Grabowski,[1] William W. Brennessel,[3] Marjorie A. Jones,[2] Michael I. Webb[1,*]

1 Department of Chemistry and Biochemistry, SUNY Geneseo, Geneseo, NY, 14454

2 Department of Chemistry, Illinois State University, Normal, IL, 61790

3 Department of Chemistry, University of Rochester, Rochester, NY, 14627

Corresponding Author: Michael I. Webb, mwebb@geneseo.edu

Table of Contents:

- **Figures S1-S10:** ^{1}H and ^{13}C NMR spectra for the prepared Ru complexes.
- **Figures S11-S15:** UV-Vis spectra for the prepared complexes in PBS at 37 °C for 8 hours.
- **Figures S16-S20:** ^{1}H spectra for the prepared Ru complexes in 33-75% DMSO-D_6 in D_2O.
- **Figures S21-S25:** ^{1}H spectra for the prepared Ru complexes (18 mM) with imidazole (18 mM) in D_2O/DMSO-D_6.
- **Figure S26:** Additional TEM images collected for all of the Ru complexes with $A\beta_{40}$ from the DLS filtrates.
- **Figure S27-S36:** Fluorescence emission spectra at various Ru-HSA ratios by the titration of HSA-DG (1:1) or HSA-WF (1:1) with each Ru complex.
- **Figures S37 & S38:** Cell viability for C6 and P12 cells with the respective Ru complexes.
- **Figure S39-S43:** Images of the C6 cells following incubation with the Ru complexes.
- **Figure S44-S48:** Images of the P12 cells following incubation with the Ru complexes.
- **Table S1:** Refinement parameters for the X-ray crystal structure of **RuPA**.
- **Table S2:** Refinement parameters for the X-ray crystal structure of **RuPMeO**.

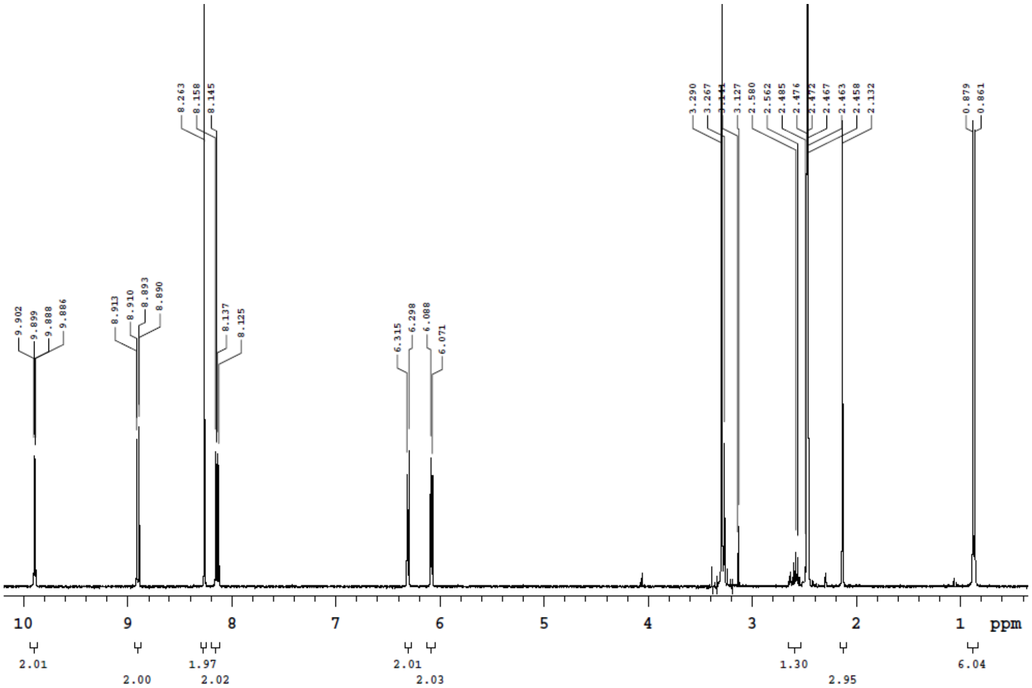

Figure S1. ^{1}H NMR spectrum of complex **RuP** in DMSO-D$_6$.

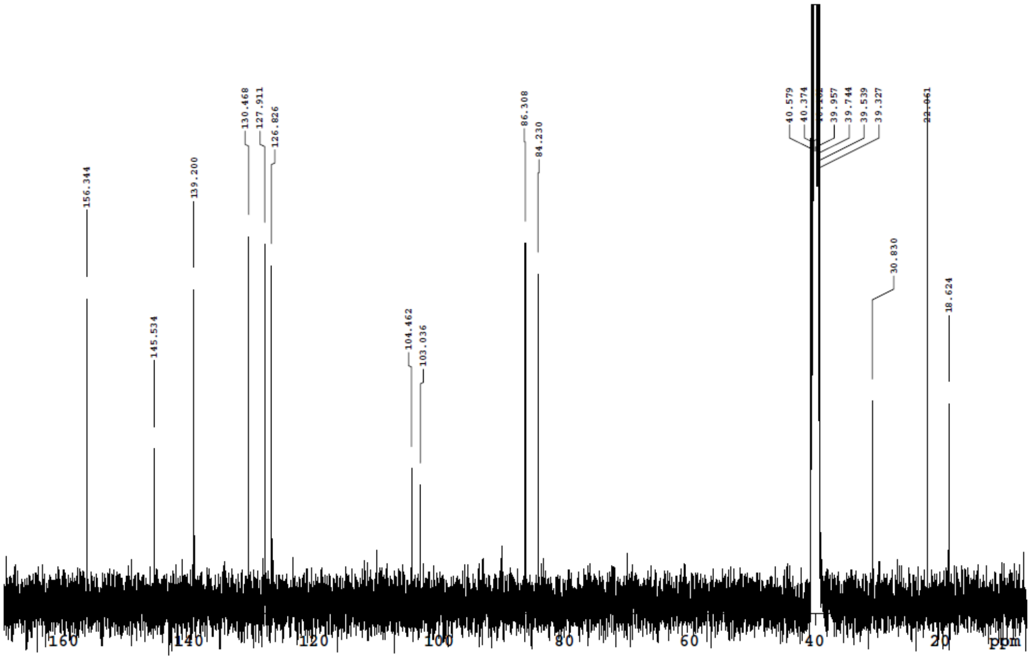

Figure S2. ^{13}C NMR spectrum of complex **RuP** in DMSO-D$_6$.

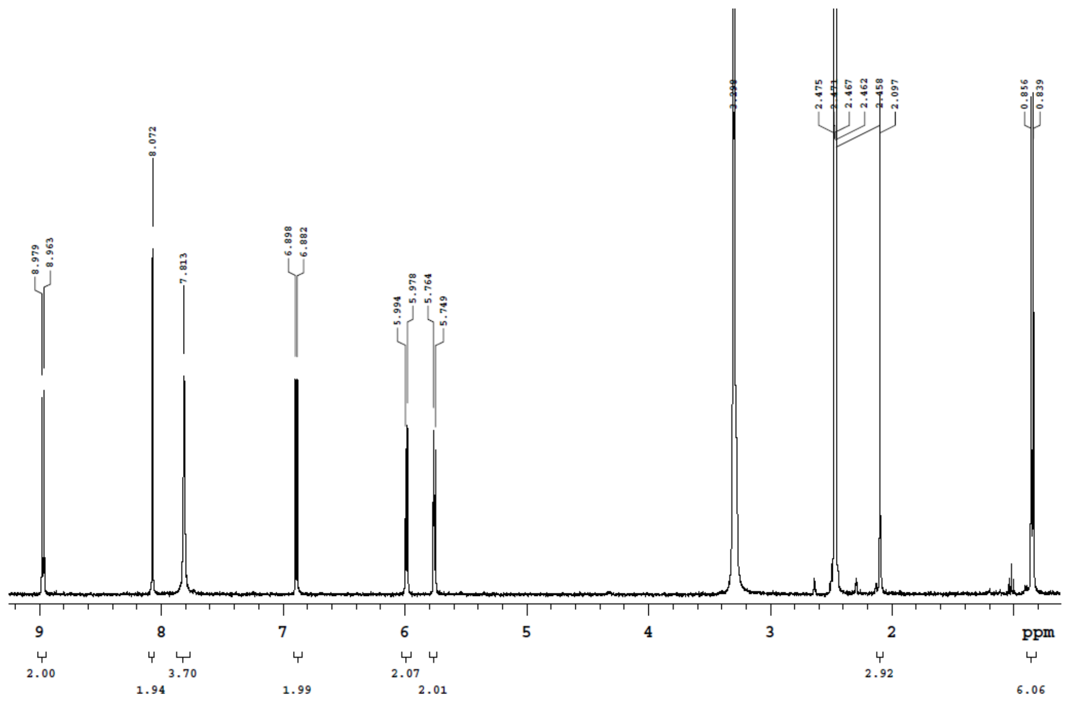

Figure S3. ^{1}H NMR spectrum of complex **RuPA** in DMSO-D_6.

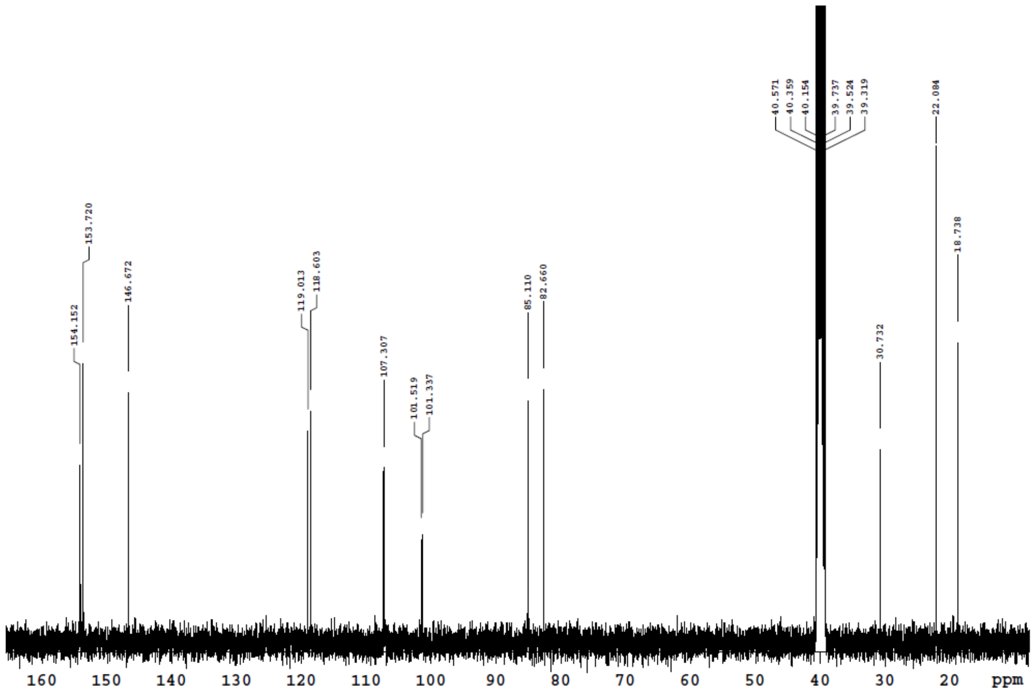

Figure S4. ^{13}C NMR spectrum of complex **RuPA** in DMSO-D_6.

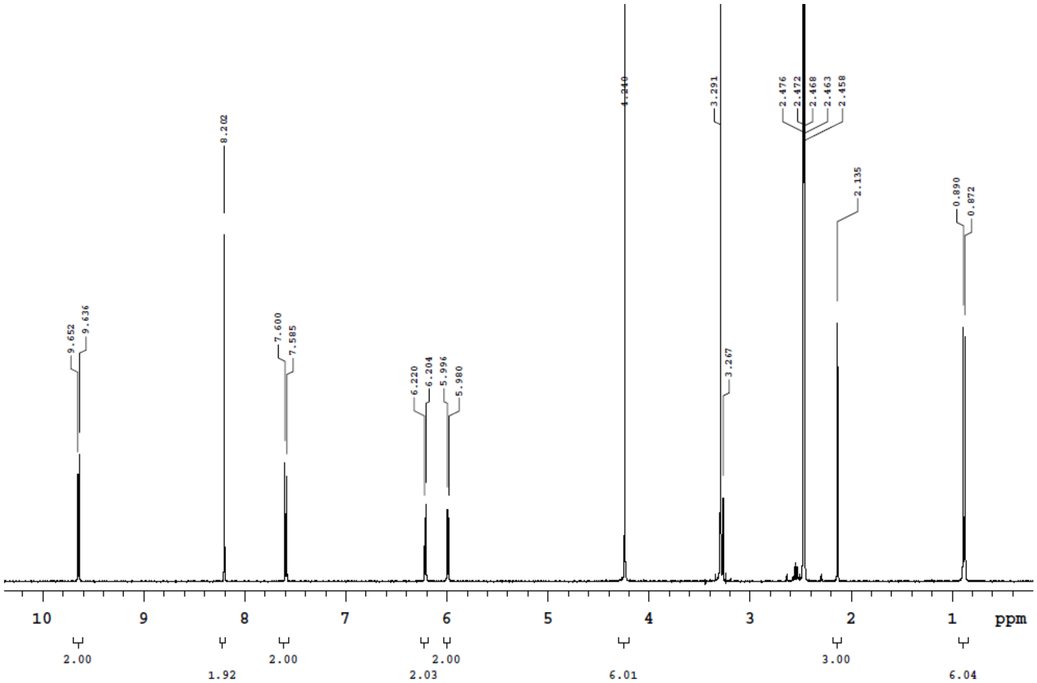

Figure S5. ^{1}H NMR spectrum of complex **RuPMeO** in DMSO-D_6.

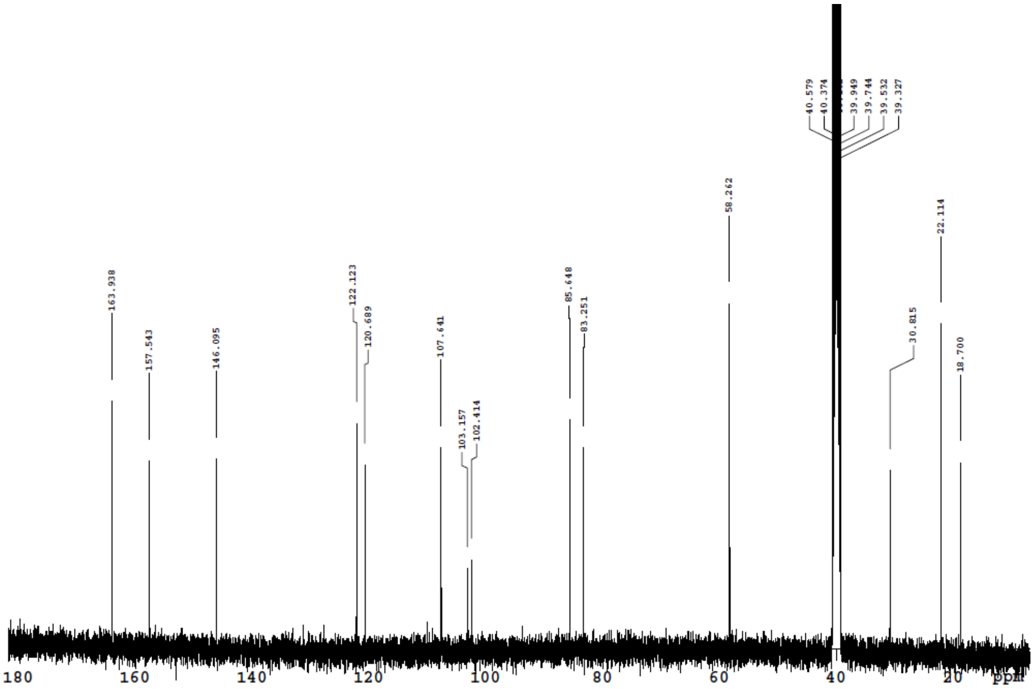

Figure S6. ^{13}C NMR spectrum of complex **RuPMeO** in DMSO-D_6.

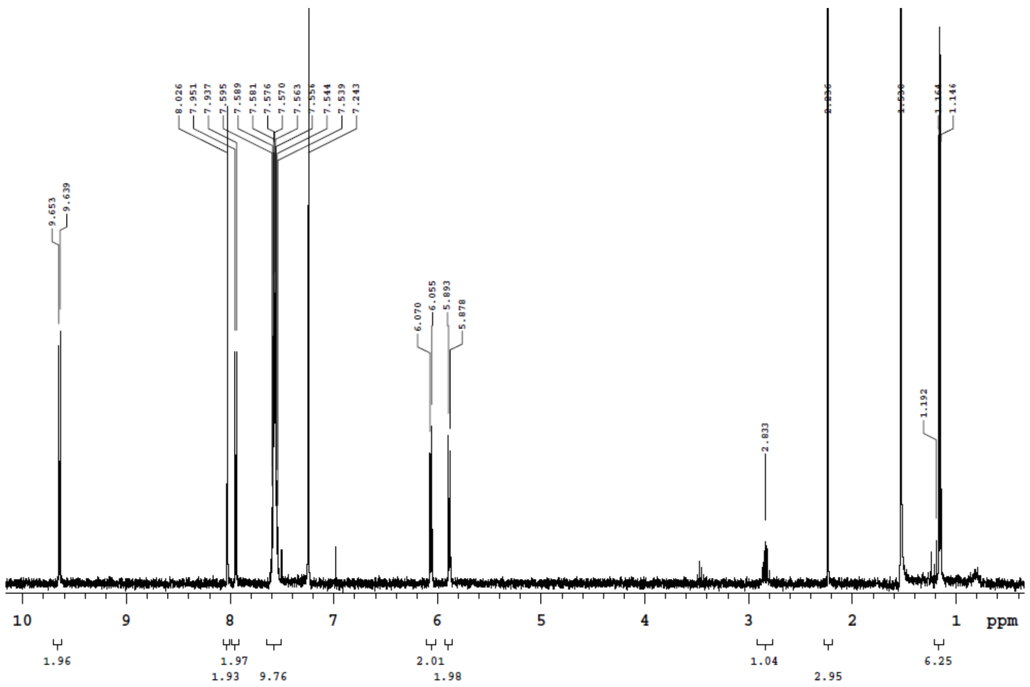

Figure S7. ^{1}H NMR spectrum of complex **RuPPh** in $CDCl_3$.

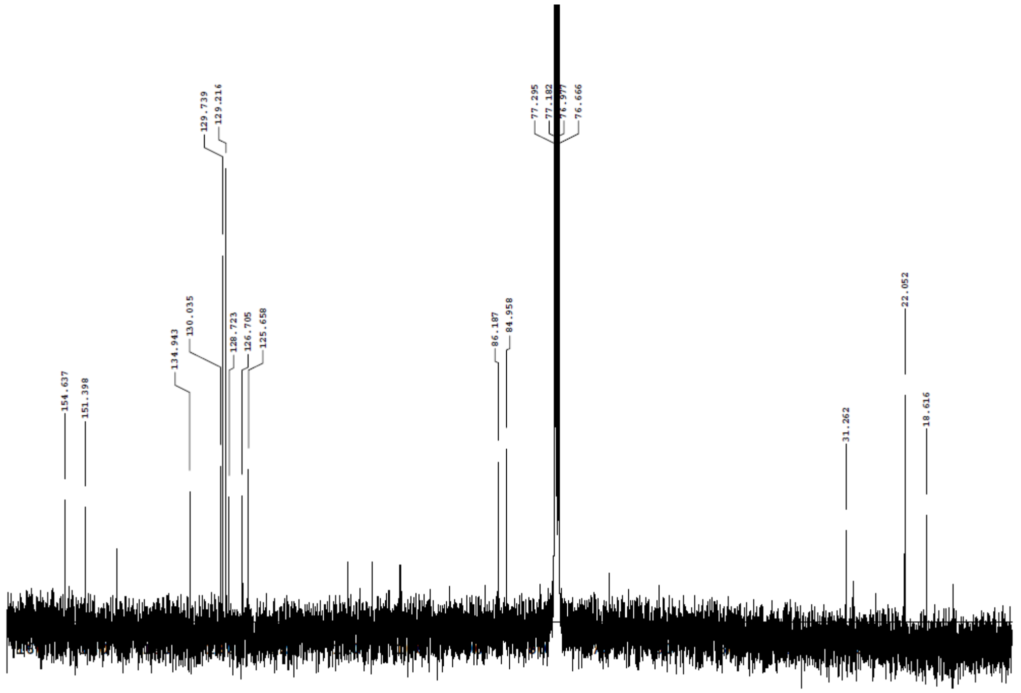

Figure S8. ^{13}C NMR spectrum of complex **RuPPh** in $CDCl_3$.

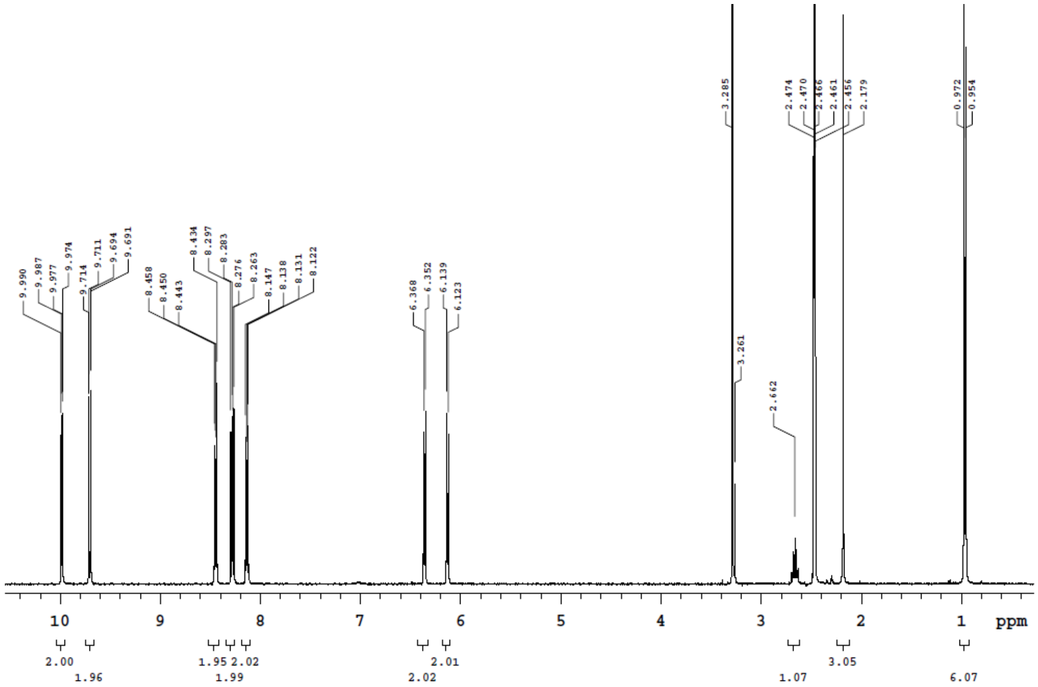

Figure S9. ^{1}H NMR spectrum of complex **RuDppz** in DMSO-D_6.

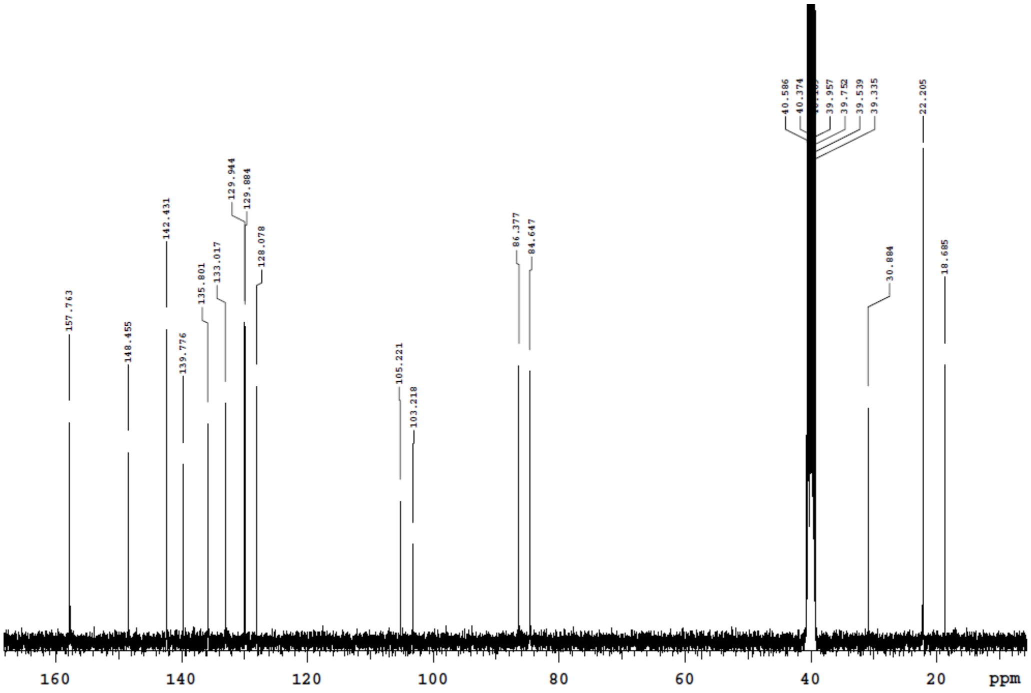

Figure S10. ^{13}C NMR spectrum of complex **RuDppz** in DMSO-D_6.

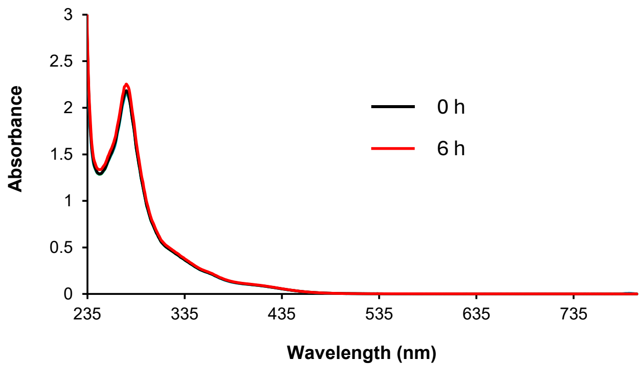

Figure S11. UV-Vis spectra of complex **RuP** (100 μM) incubated in PBS (pH 7.4) at 37 °C for up to 6 hours.

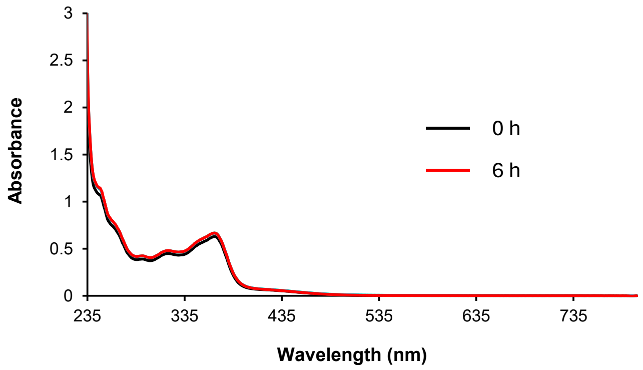

Figure S12. UV-Vis spectra of complex **RuPA** (100 μM) incubated in PBS (pH 7.4) at 37 °C for up to 6 hours.

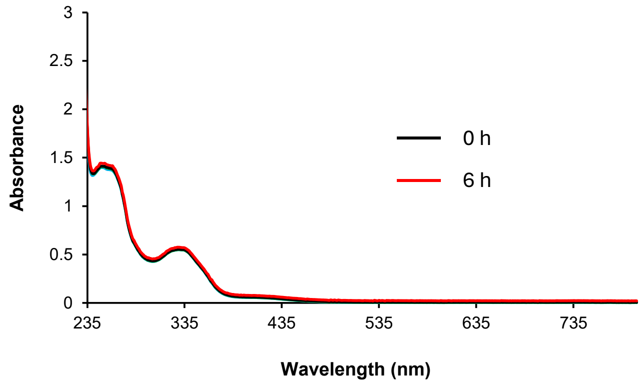

Figure S13. UV-Vis spectra of complex **RuPMeO** (100 μM) incubated in PBS (pH 7.4) at 37 °C for up to 6 hours.

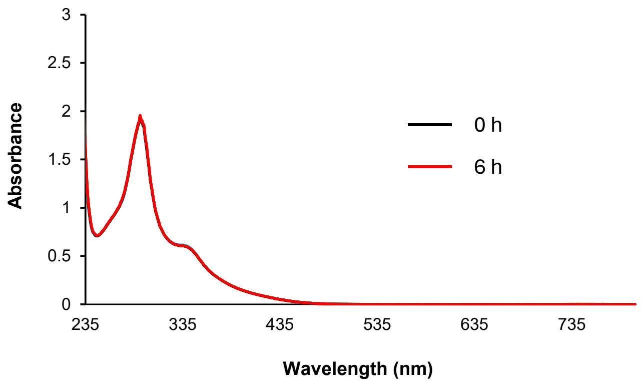

Figure S14. UV-Vis spectra of complex **RuPPh** (100 µM) incubated in PBS (pH 7.4) at 37 °C for up to 6 hours.

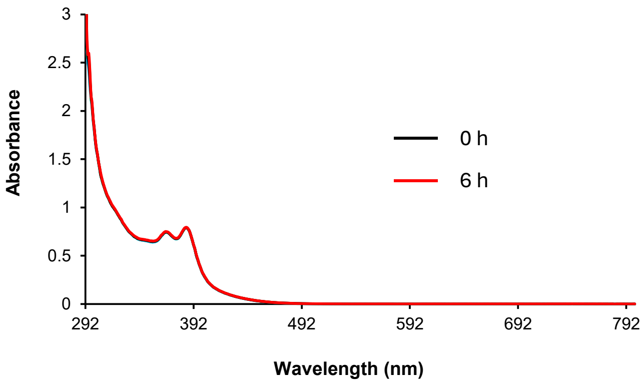

Figure S15. UV-Vis spectra of complex **RuDppz** (100 µM) incubated in PBS (pH 7.4) at 37 °C for up to 6 hours.

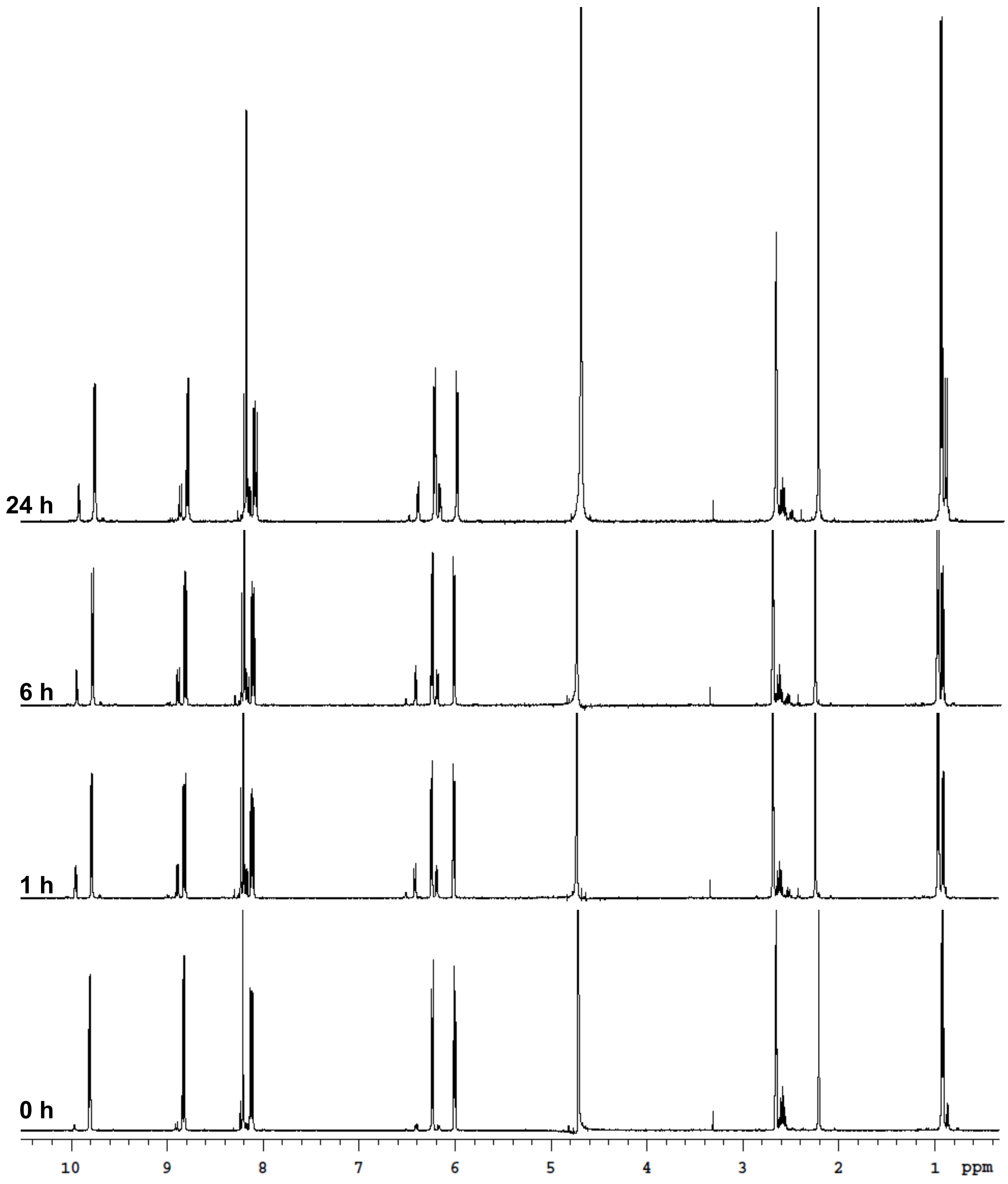

Figure S16. ^{1}H NMR spectrum of complex **RuP** in 33% DMSO-D_6 and D_2O over 24 hours.

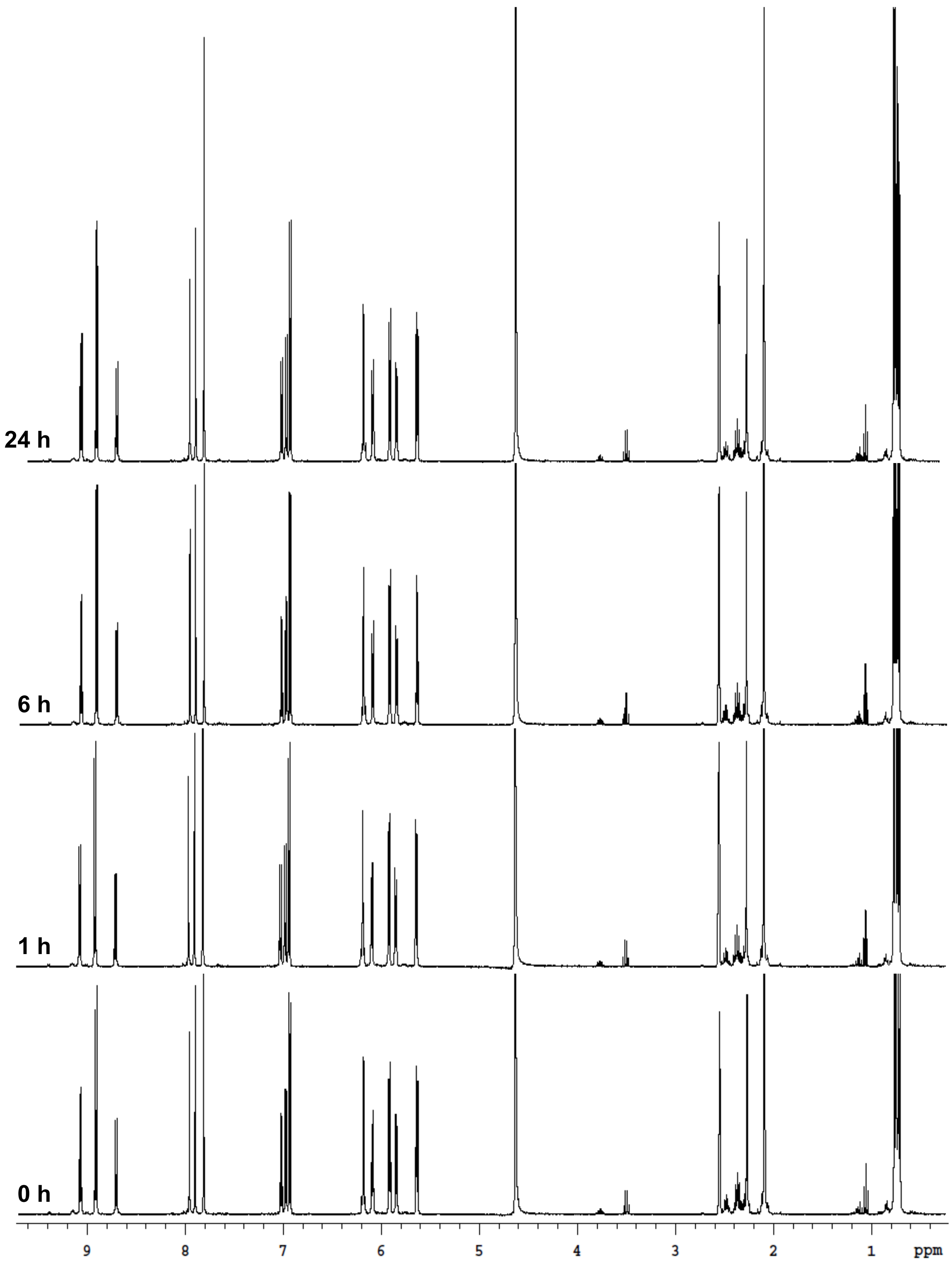

Figure S17. ^{1}H NMR spectrum of complex **RuPA** in 33% DMSO-D_6 and D_2O over 24 hours.

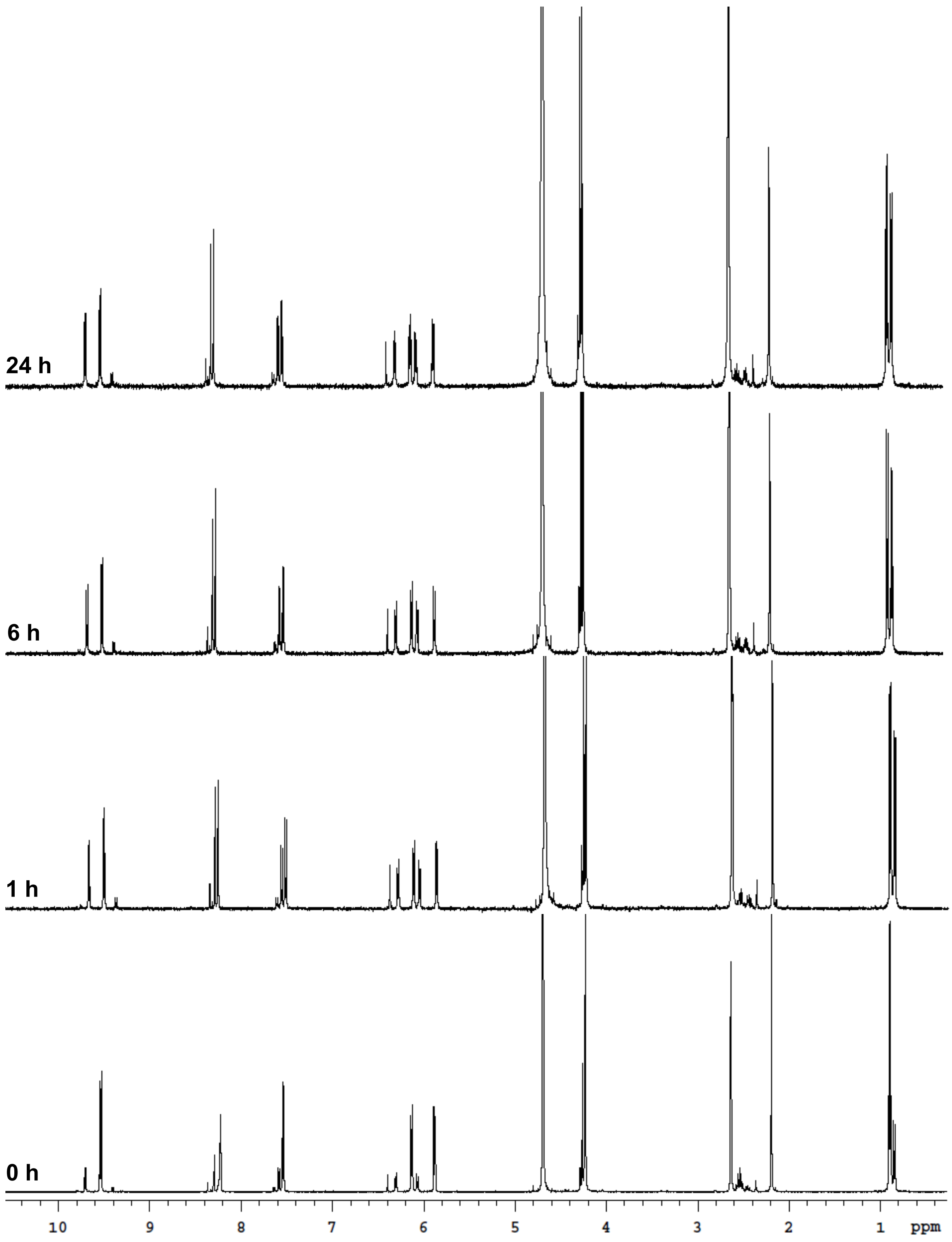

Figure S18. ^{1}H NMR spectrum of complex **RuPMeO** in 33% DMSO-D_6 and D_2O over 24 hours.

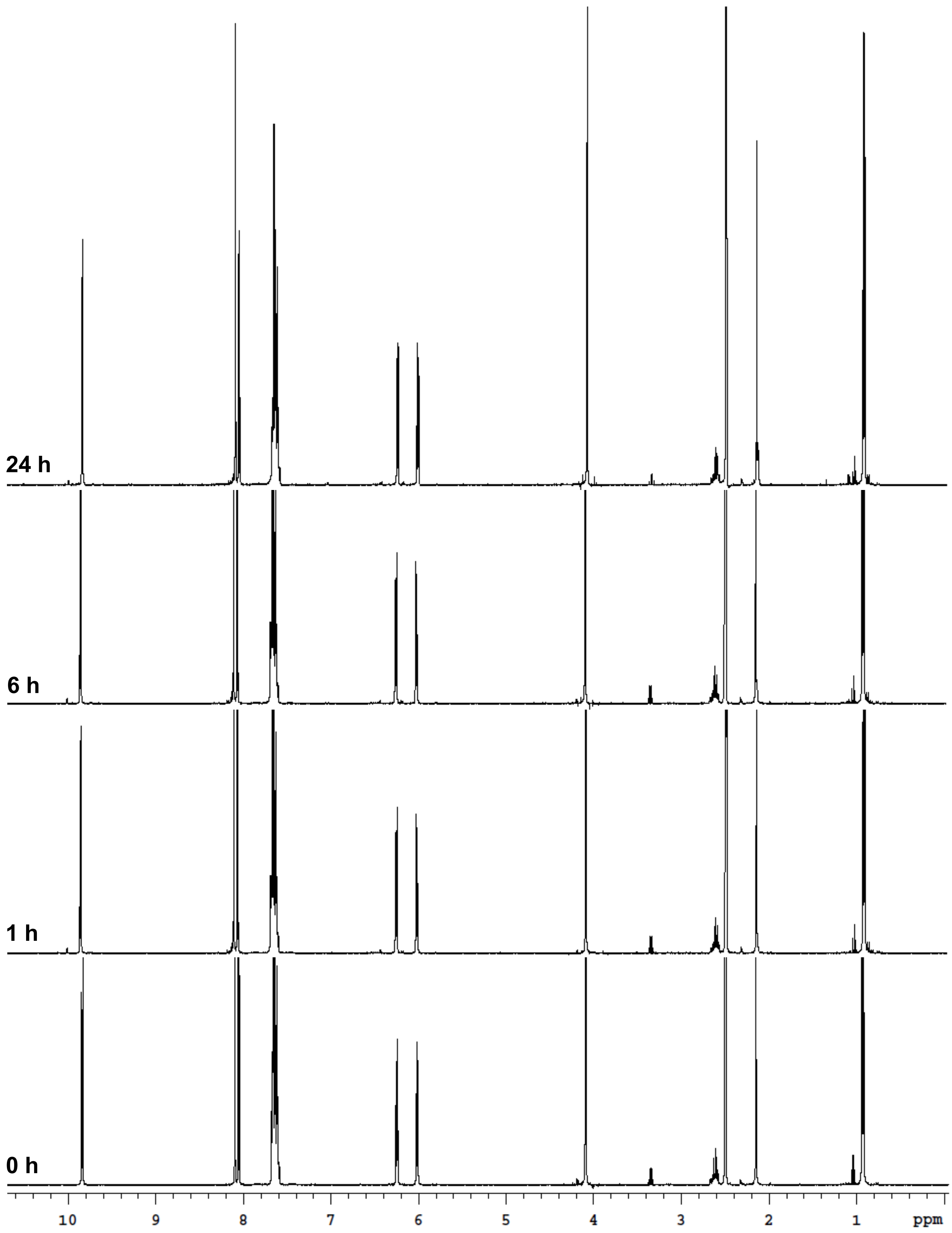

Figure S19. ^{1}H NMR spectrum of complex **RuPPh** in 75% DMSO-D_6 and D_2O over 24 hours.

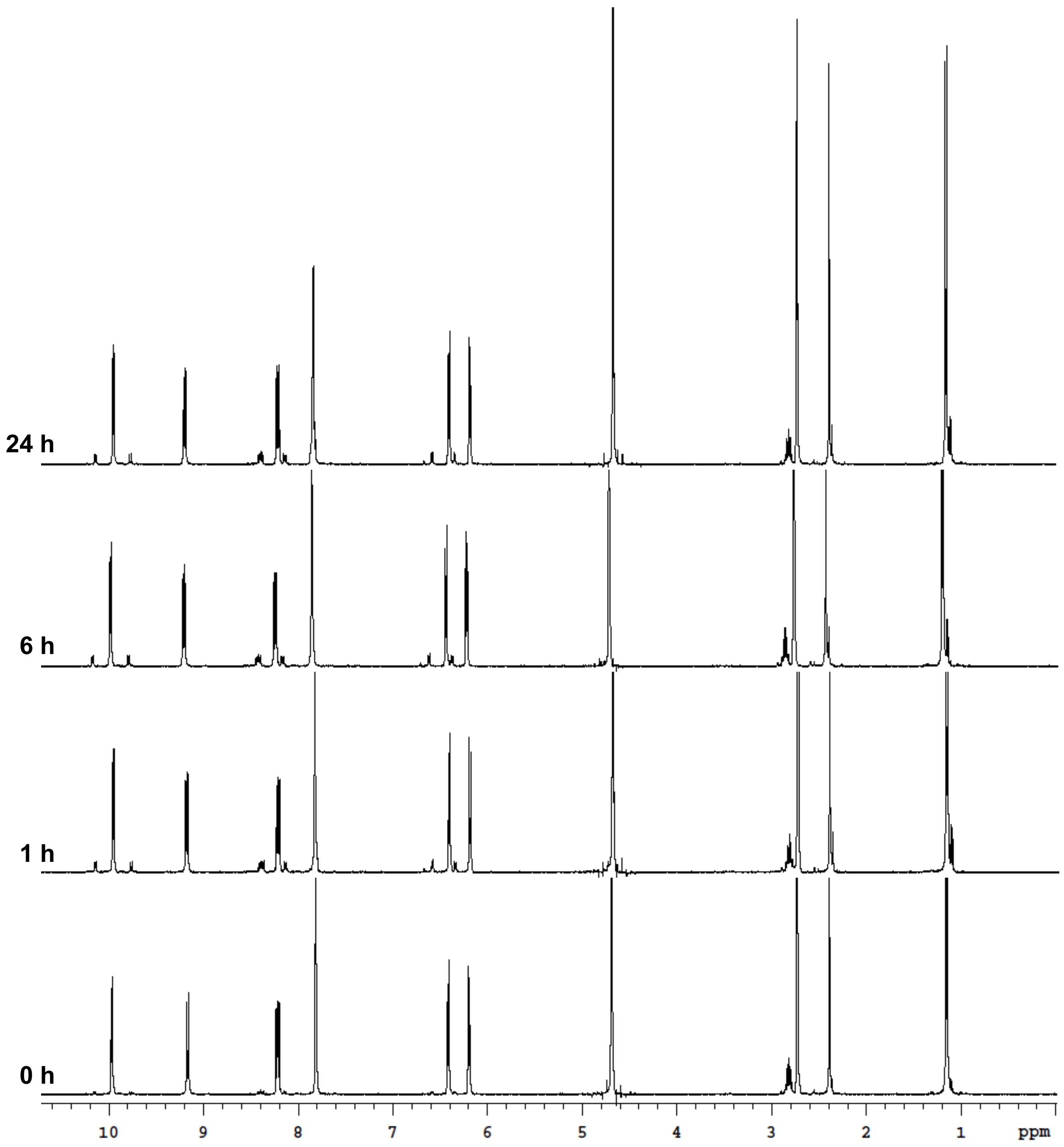

Figure S20. ^{1}H NMR spectrum of complex **RuDppz** in 50% DMSO-D_6 and D_2O over 24 hours.

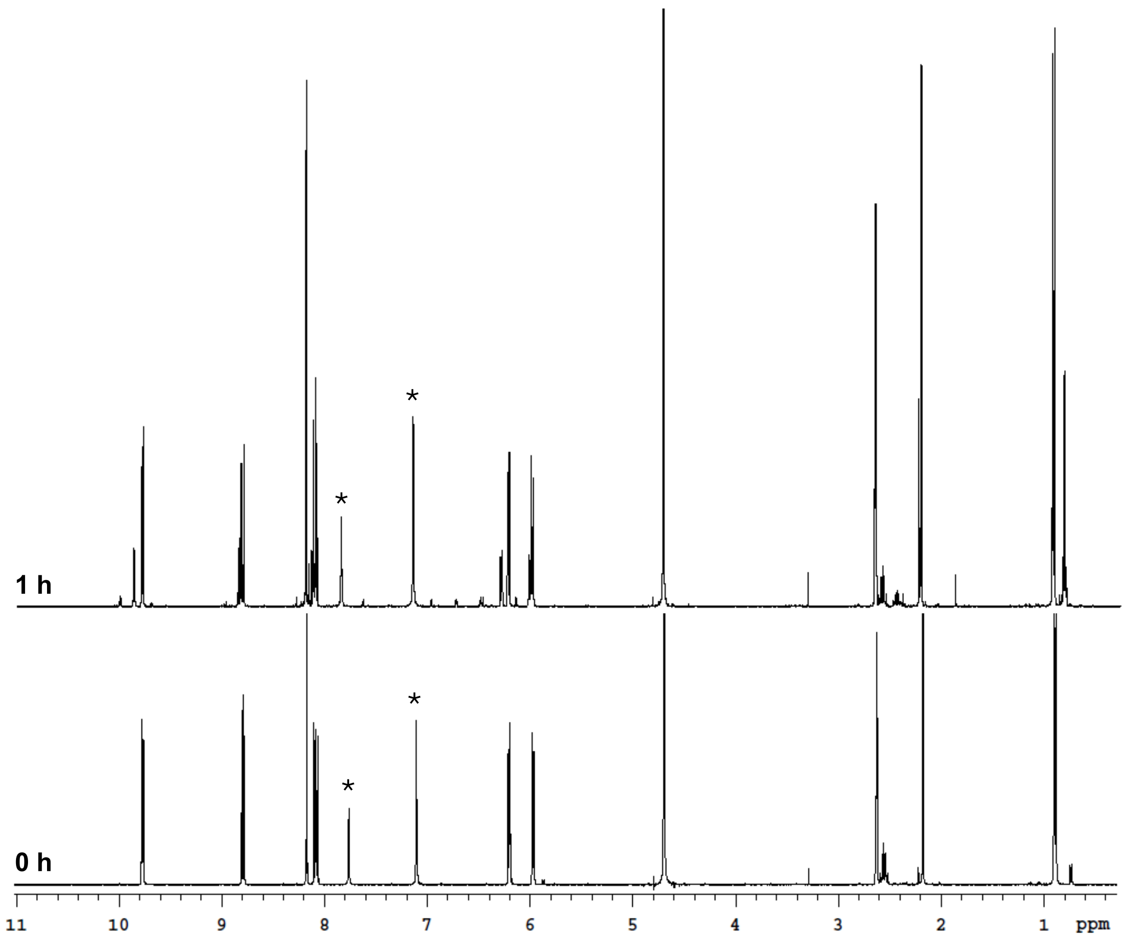

Figure S21. ^1H NMR spectra in 33% DMSO-D_6 and D_2O of complex **RuP** (18 mM) with imidazole (18 mM). The bottom spectrum is immediately after mixing, while the top is after 1 hour of incubation at 37 °C. The free imidazole peaks are marked with an asterisk (*).

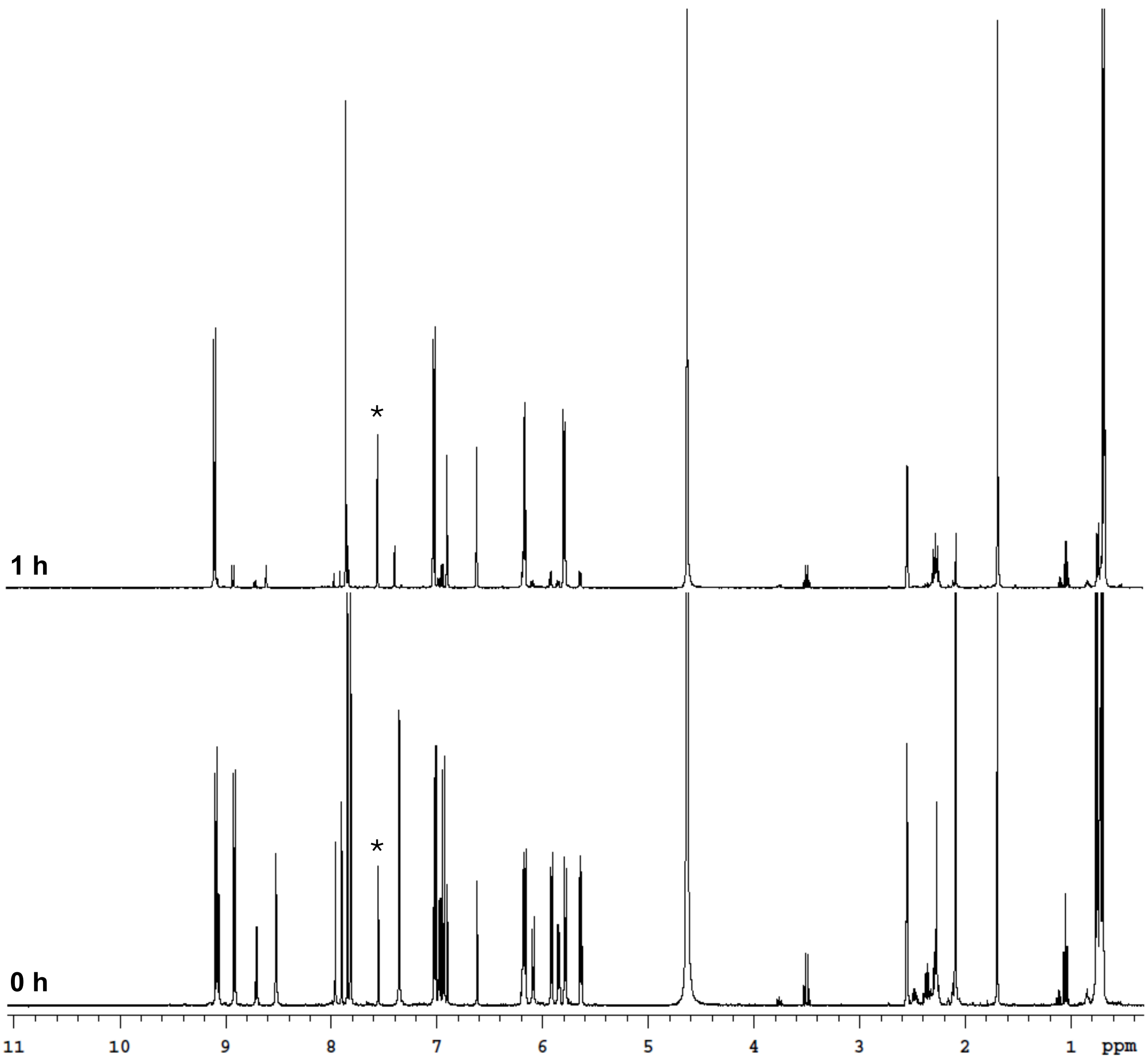

Figure S22. ^{1}H NMR spectra in 33% DMSO-D_6 and D_2O of complex **RuPA** (18 mM) with imidazole (18 mM). The bottom spectrum is immediately after mixing, while the top is after 1 hour of incubation at 37 °C. The free visible imidazole peak is marked with an asterisk (*).

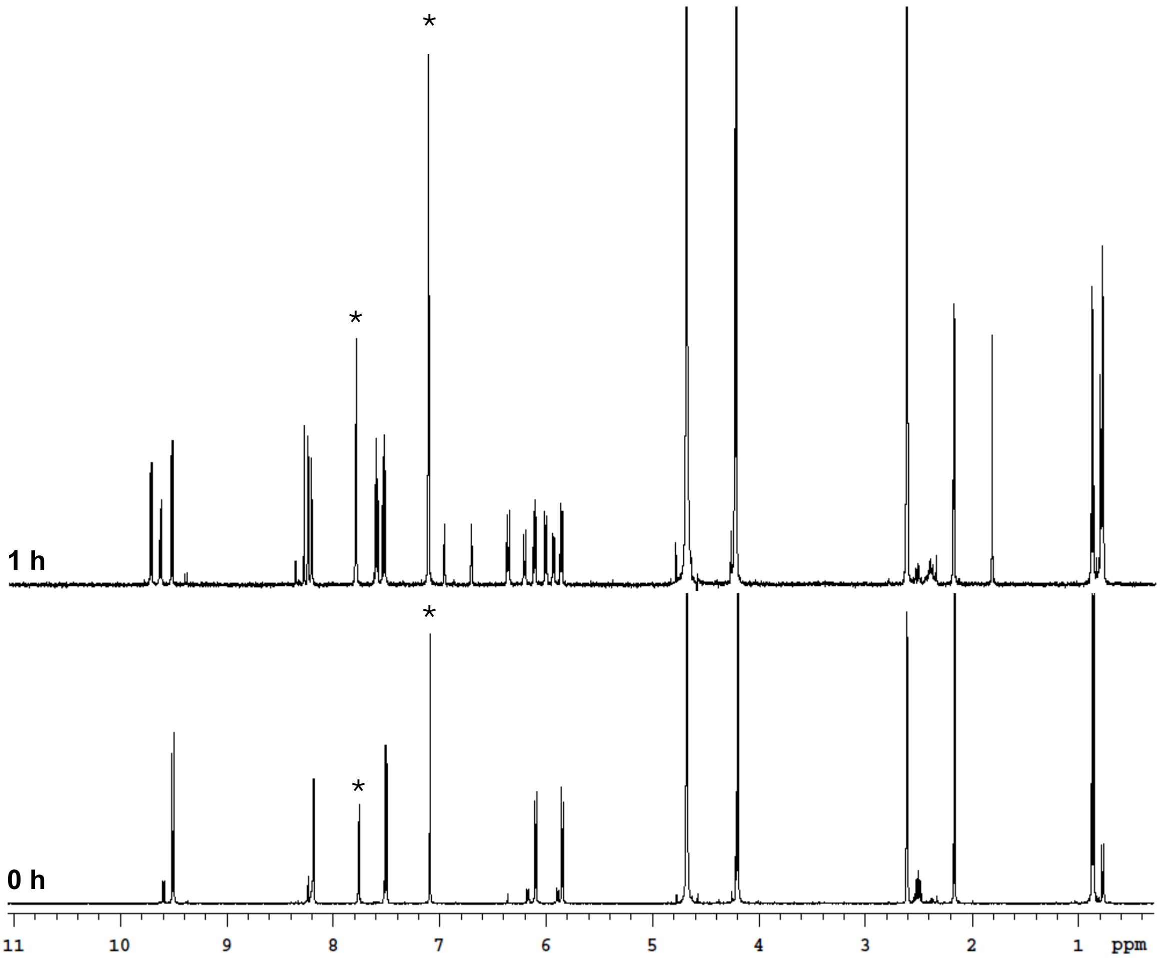

Figure S23. ^{1}H NMR spectra in 33% DMSO-D_6 and D_2O of complex **RuPMeO** (18 mM) with imidazole (18 mM). The bottom spectrum is immediately after mixing, while the top is after 1 hour of incubation at 37 °C. The free imidazole peaks are marked with an asterisk (*).

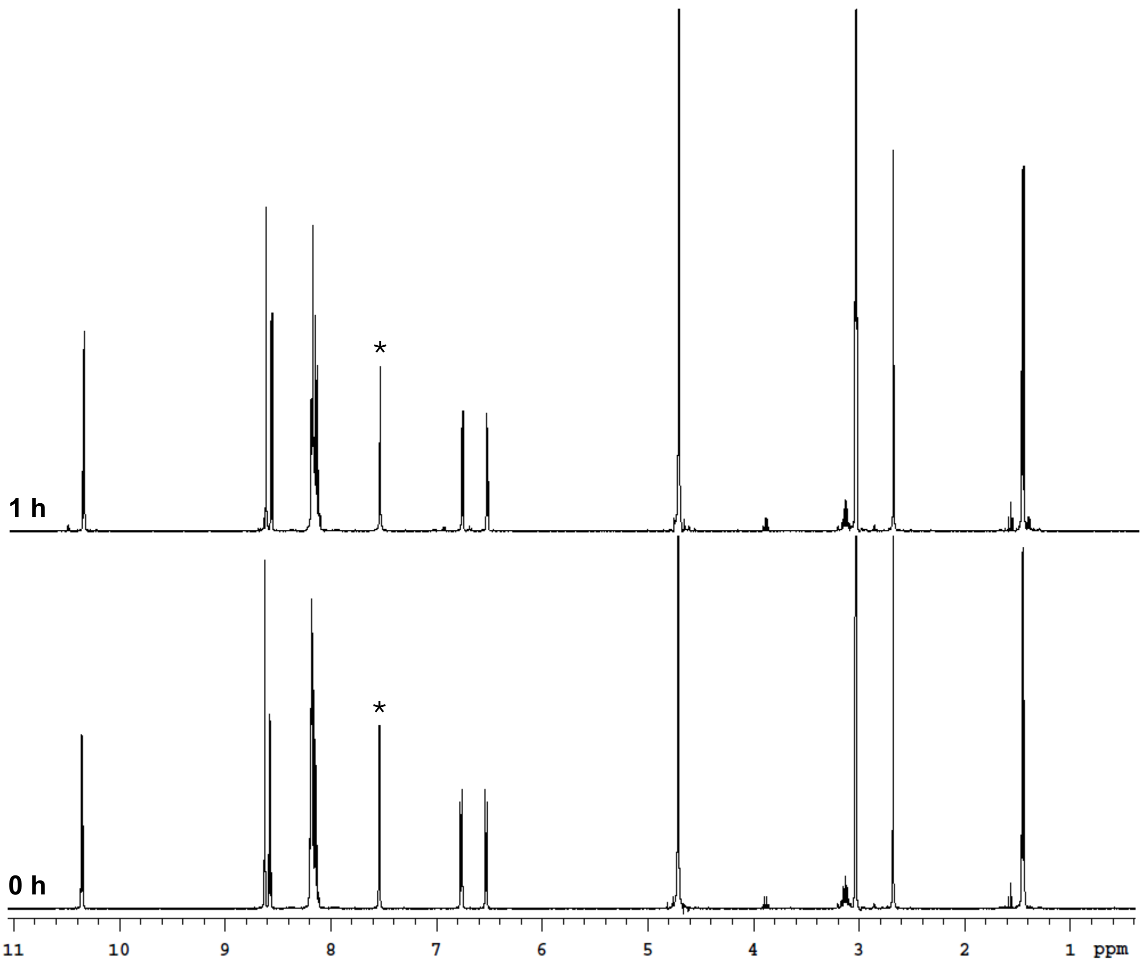

Figure S24. ^{1}H NMR spectra in 75% DMSO-D_6 and D_2O of complex **RuPPh** (18 mM) with imidazole (18 mM). The bottom spectrum is immediately after mixing, while the top is after 1 hour of incubation at 37 °C. The free visible imidazole peak is marked with an asterisk (*).

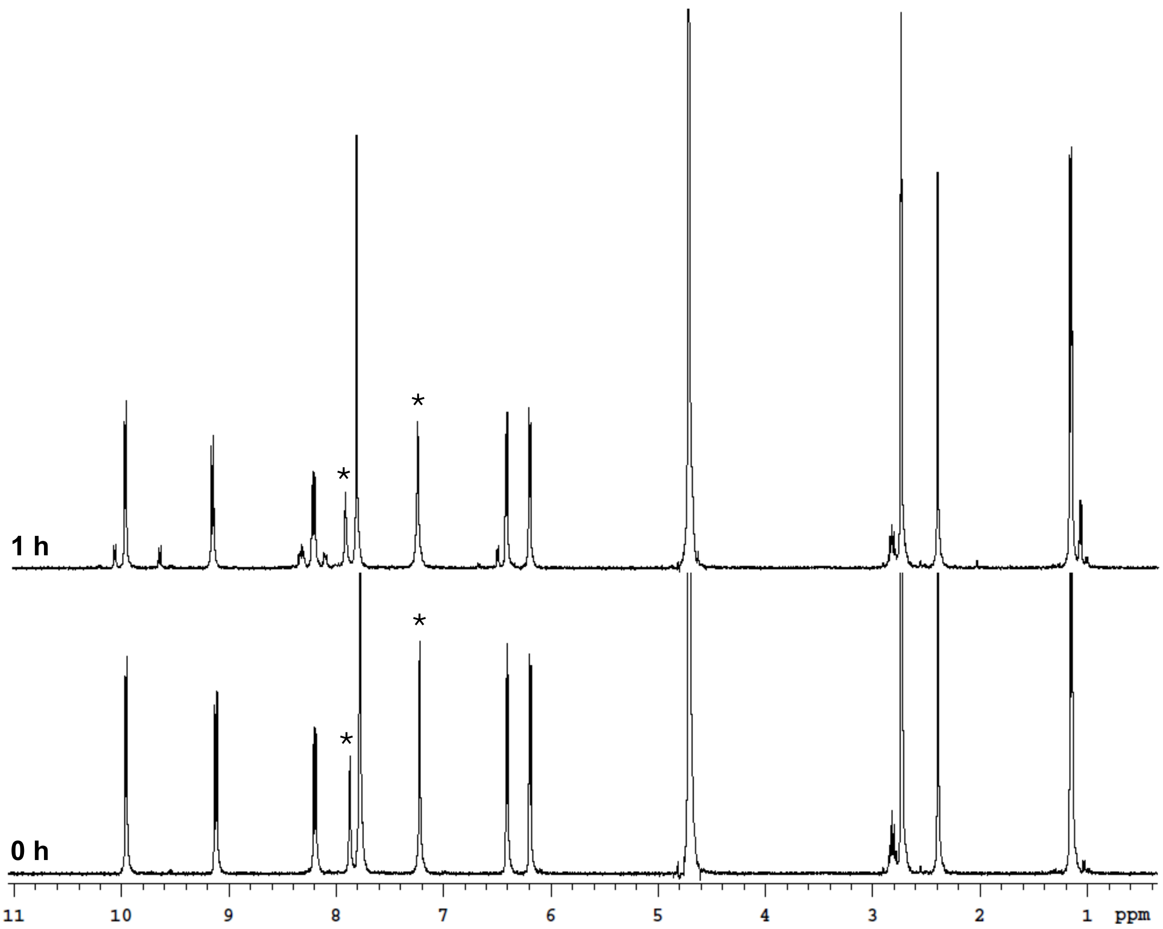

Figure S25. ^{1}H NMR spectra in 33% DMSO-D_6 and D_2O of complex **RuDppz** (18 mM) with imidazole (18 mM). The bottom spectrum is immediately after mixing, while the top is after 1 hour of incubation at 37 °C. The free imidazole peaks are marked with an asterisk (*).

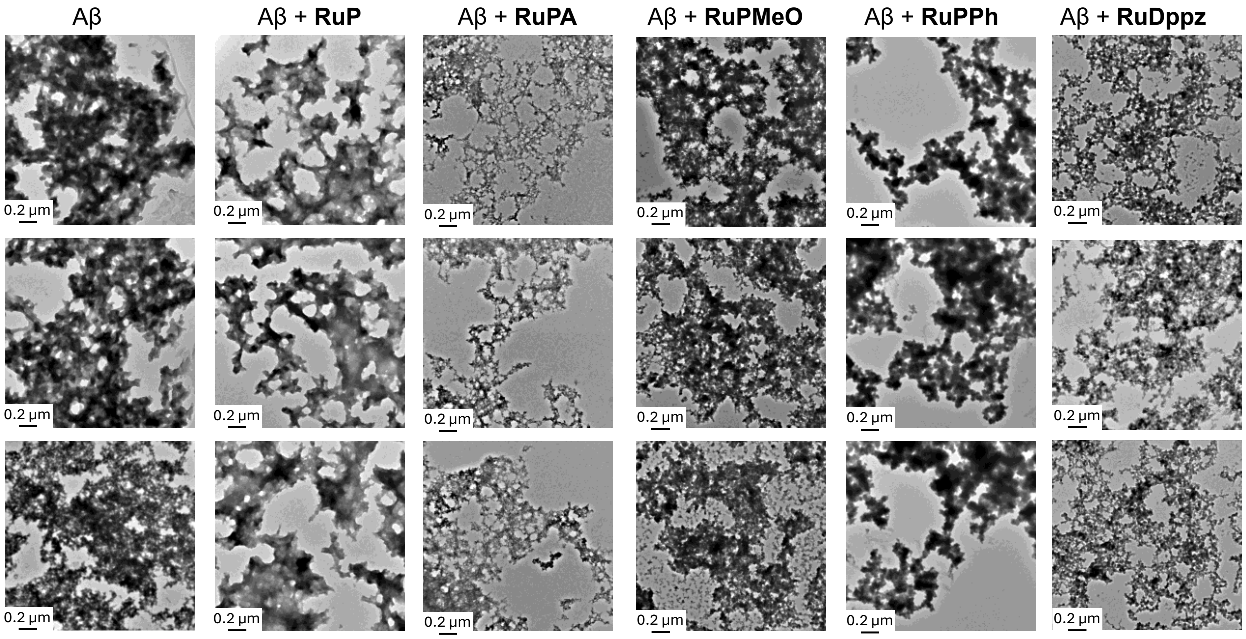

Figure S26. Additional TEM images collected for all of the Ru complexes with $A\beta_{40}$ from the DLS filtrates. Scale bars are provided within each image.

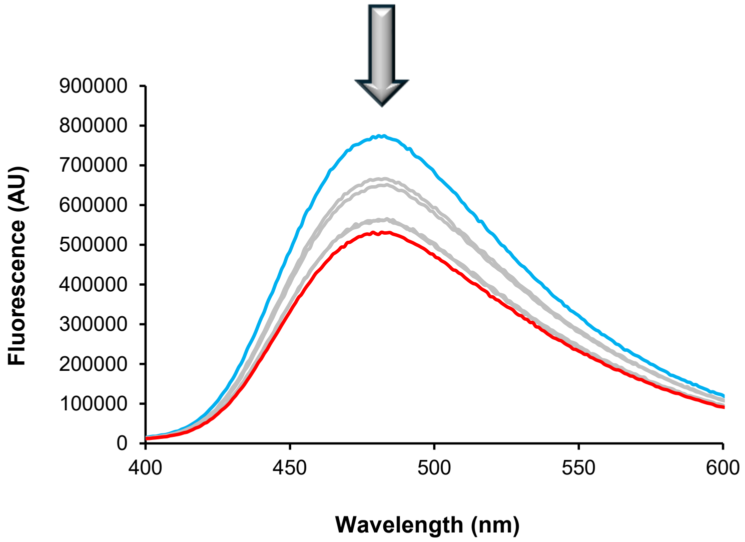

Figure S27. Fluorescence emission spectra at various Ru-HSA ratios by the titration of HSA-DG (1:1) with **RuP**. Experimental conditions: λ_{ex} = 330 nm, λ_{em} = 350-600 nm, [HSA] = [DG] = 2.5 μM, [Ru] = 0 (blue line), 12.5, 25, 32.5, 50, and 62.5 μM (red line), in PBS (pH 7.4).

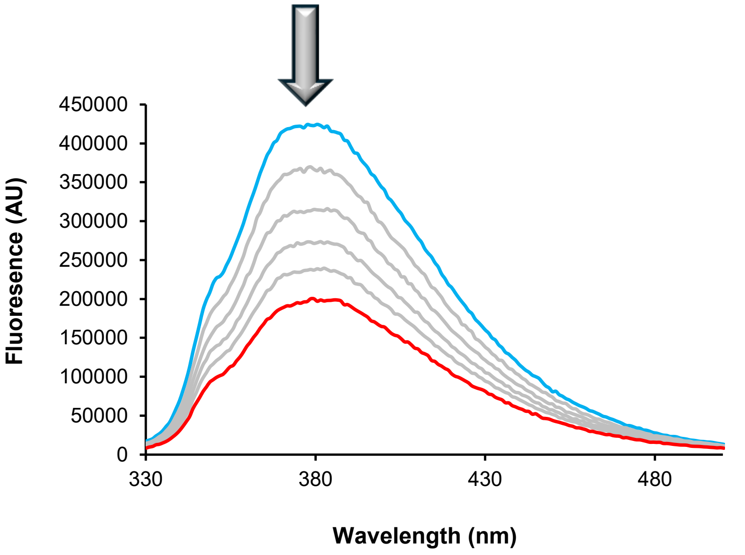

Figure S28. Fluorescence emission spectra at various Ru-HSA ratios by the titration of HSA-WF (1:1) with **RuP**. Experimental conditions: λ_{ex} = 295 nm, λ_{em} = 330-500 nm, [HSA] = [WF] = 2.5 μM, [Ru] = 0 (blue line), 5, 10, 15, 20, and 25 μM (red line), in PBS (pH 7.4).

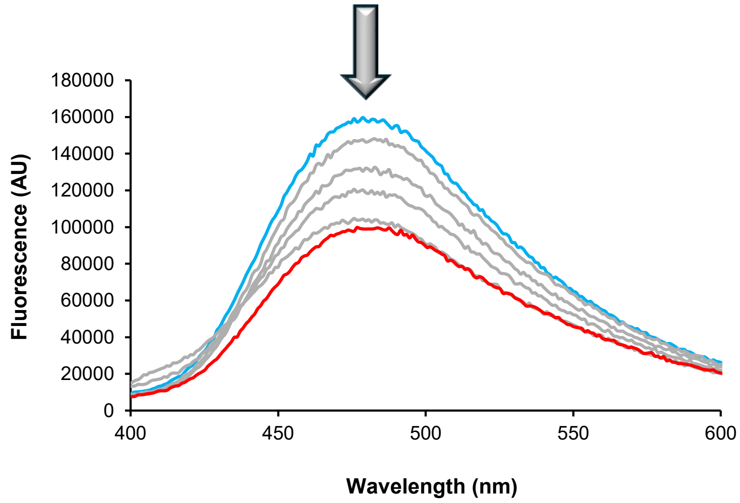

Figure S29. Fluorescence emission spectra at various Ru-HSA ratios by the titration of HSA-DG (1:1) with **RuPA**. Experimental conditions: λ_{ex} = 330 nm, λ_{em} = 350-600 nm, [HSA] = [DG] = 2.5 μM, [Ru] = 0 (blue line), 12.5, 25, 32.5, 50, and 62.5 μM (red line), in PBS (pH 7.4).

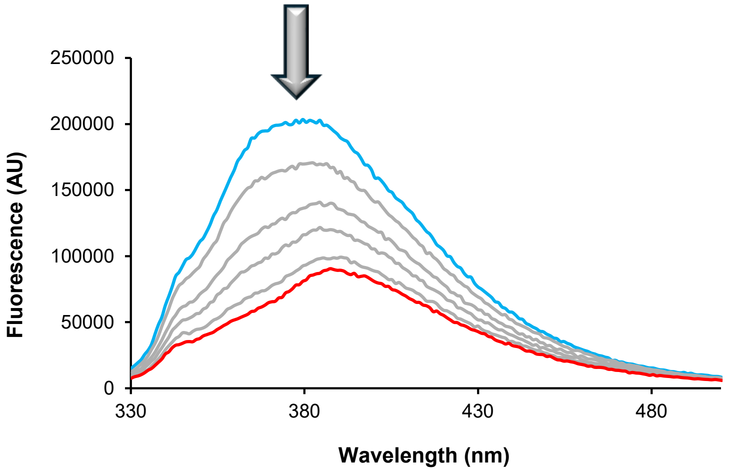

Figure S30. Fluorescence emission spectra at various Ru-HSA ratios by the titration of HSA-WF (1:1) with **RuPA**. Experimental conditions: λ_{ex} = 295 nm, λ_{em} = 330-500 nm, [HSA] = [WF] = 2.5 μM, [Ru] = 0 (blue line), 12.5, 25, 32.5, 50, and 62.5 μM (red line), in PBS (pH 7.4).

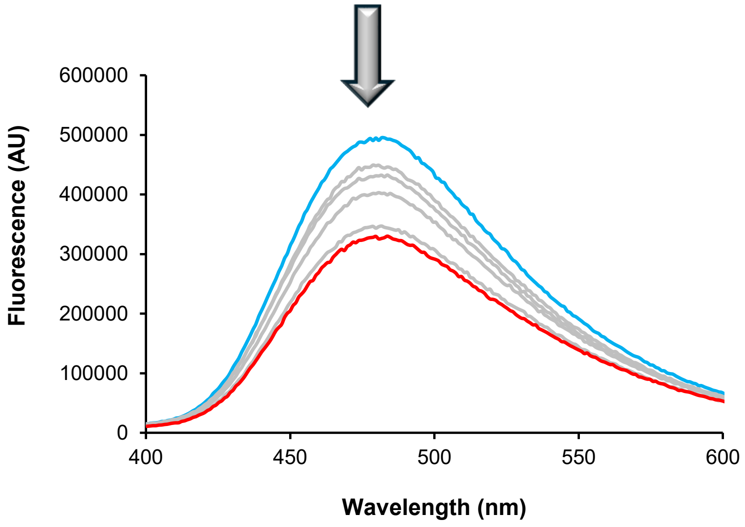

Figure S31. Fluorescence emission spectra at various Ru-HSA ratios by the titration of HSA-DG (1:1) with **RuBMeO**. Experimental conditions: λ_{ex} = 330 nm, λ_{em} = 350-600 nm, [HSA] = [DG] = 2.5 μM, [Ru] = 0 (blue line), 12.5, 25, 32.5, 50, and 62.5 μM (red line), in PBS (pH 7.4).

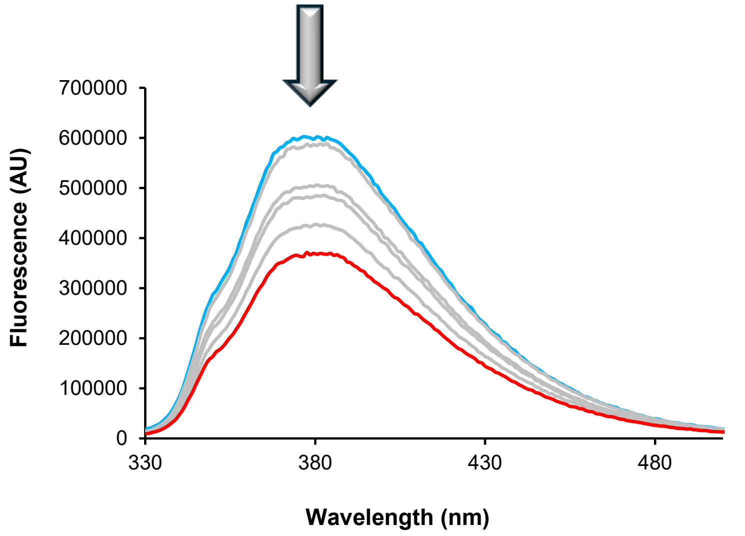

Figure S32. Fluorescence emission spectra at various Ru-HSA ratios by the titration of HSA-WF (1:1) with **RuBMeO**. Experimental conditions: λ_{ex} = 295 nm, λ_{em} = 330-500 nm, [HSA] = [WF] = 2.5 μM, [Ru] = 0 (blue line), 12.5, 25, 32.5, 50, and 62.5 μM (red line), in PBS (pH 7.4).

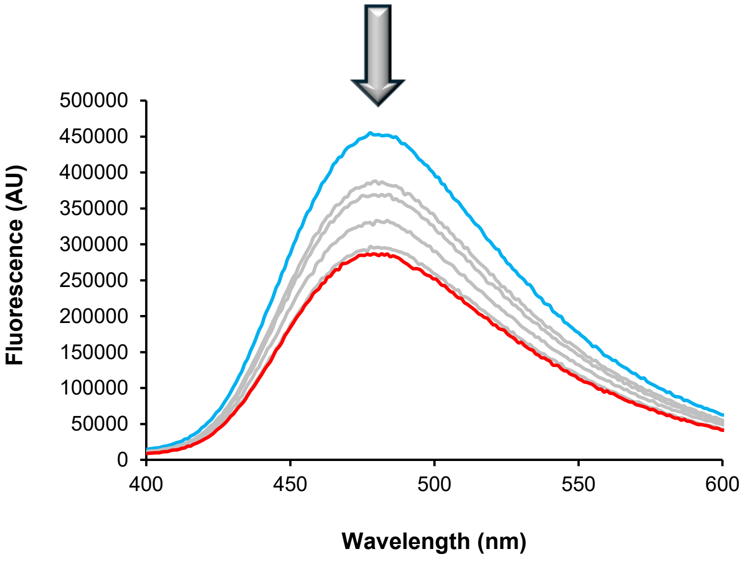

Figure S33. Fluorescence emission spectra at various Ru-HSA ratios by the titration of HSA-DG (1:1) with **RuPPh**. Experimental conditions: λ_{ex} = 330 nm, λ_{em} = 350-600 nm, [HSA] = [DG] = 2.5 μM, [Ru] = 0 (blue line), 12.5, 25, 32.5, 50, and 62.5 μM (red line), in PBS (pH 7.4).

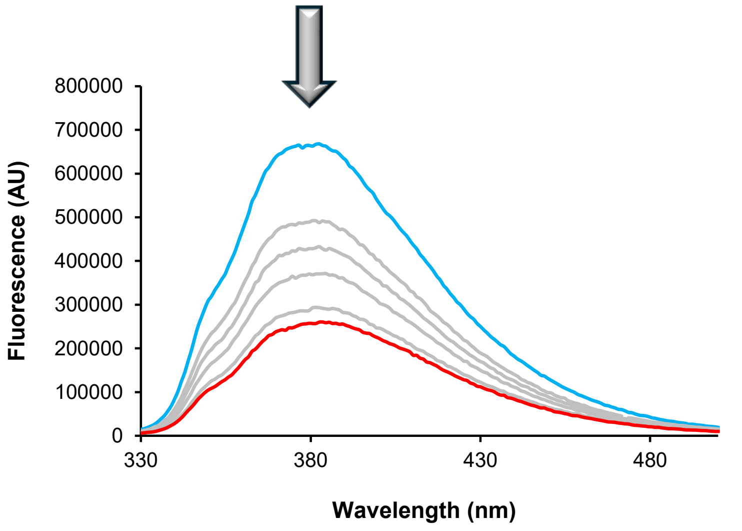

Figure S34. Fluorescence emission spectra at various Ru-HSA ratios by the titration of HSA-WF (1:1) with **RuPPh**. Experimental conditions: λ_{ex} = 295 nm, λ_{em} = 330-500 nm, [HSA] = [WF] = 2.5 μM, [Ru] = 0 (blue line), 12.5, 25, 32.5, 50, and 62.5 μM (red line), in PBS (pH 7.4).

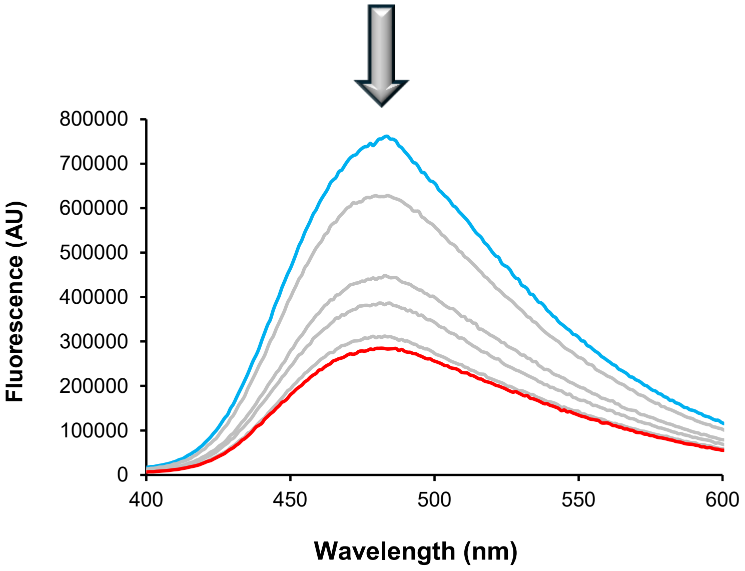

Figure S35. Fluorescence emission spectra at various Ru-HSA ratios by the titration of HSA-DG (1:1) with **RuDppz**. Experimental conditions: λ_{ex} = 330 nm, λ_{em} = 350-600 nm, [HSA] = [DG] = 2.5 μM, [Ru] = 0 (blue line), 12.5, 25, 32.5, 50, and 62.5 μM (red line), in PBS (pH 7.4).

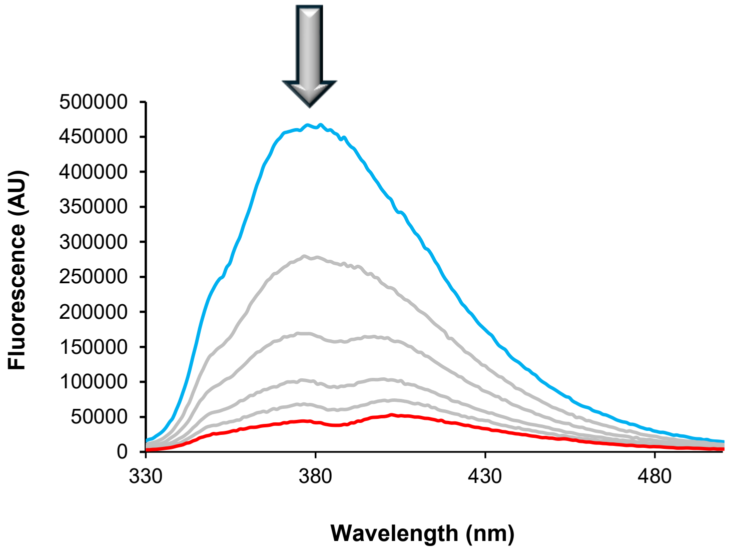

Figure S36. Fluorescence emission spectra at various Ru-HSA ratios by the titration of HSA-WF (1:1) with **RuDppz**. Experimental conditions: λ_{ex} = 295 nm, λ_{em} = 330-500 nm, [HSA] = [WF] = 2.5 μM, [Ru] = 0 (blue line), 12.5, 25, 32.5, 50, and 62.5 μM (red line), in PBS (pH 7.4).

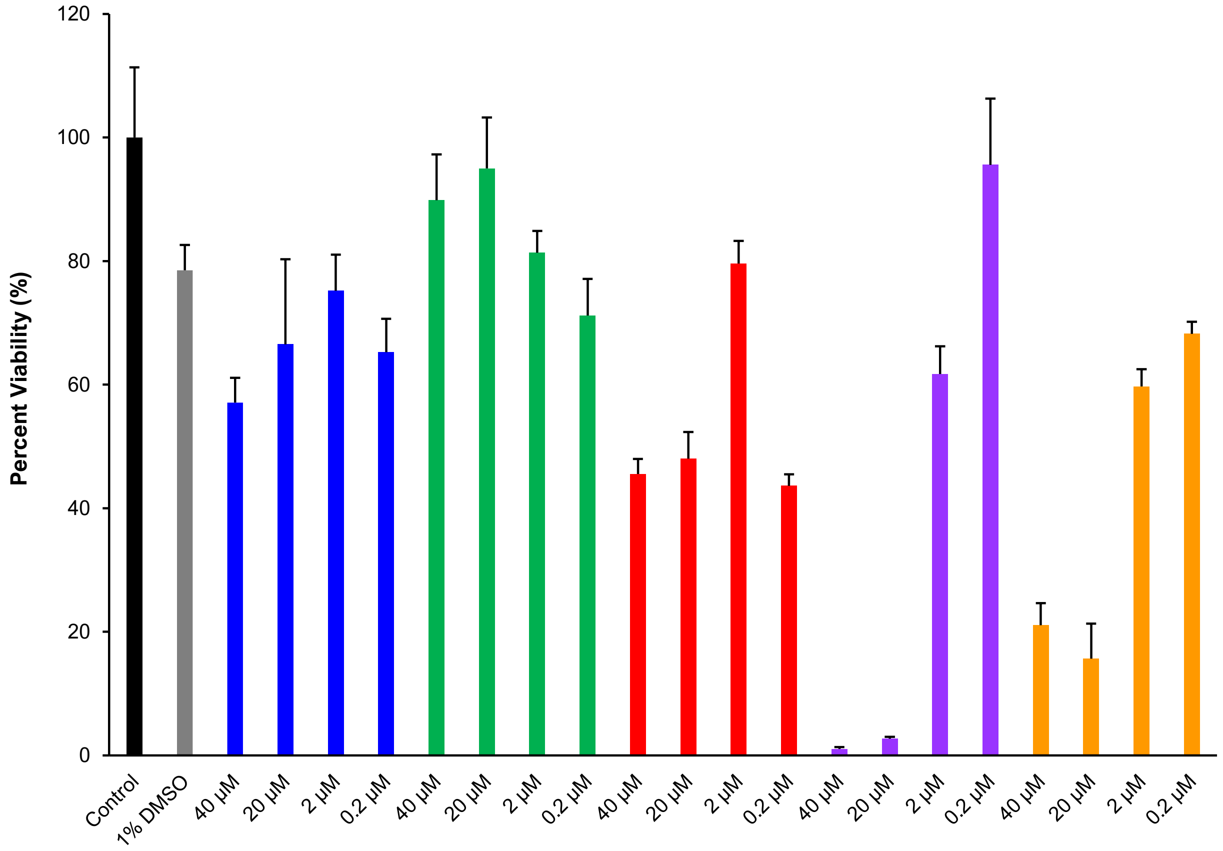

Figure S37. Cell viability of C6 cells as determined by MTT following incubation with the respective Ru complexes, where untreated cells are black, 1% DMSO is gray, **RuP** is blue, **RuPA** is green, **RuPMeO** is red, **RuPPh** is purple, and **RuDppz** is orange.

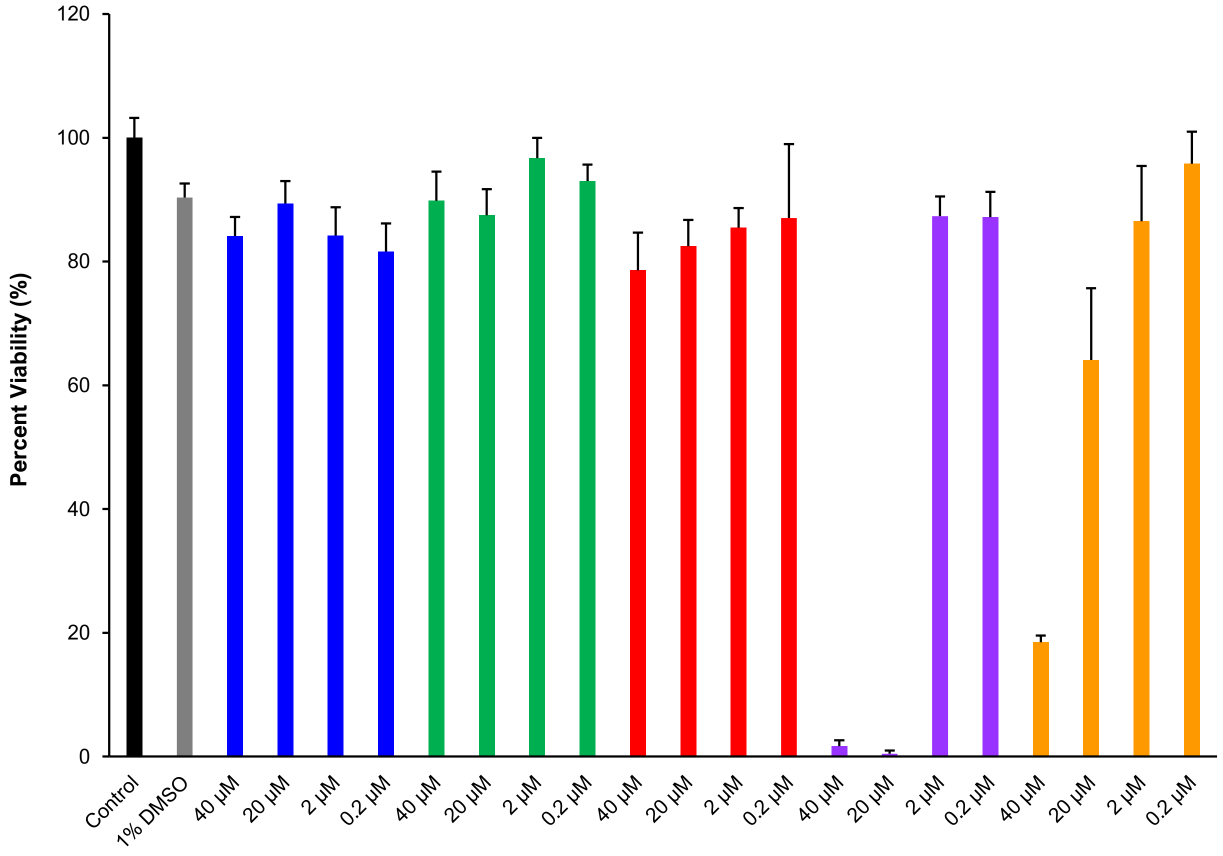

Figure S38. Cell viability of P12 cells as determined by MTT following incubation with the respective Ru complexes, where untreated cells are black, 1% DMSO is gray, **RuP** is blue, **RuPA** is green, **RuPMeO** is red, **RuPPh** is purple, and **RuDppz** is orange.

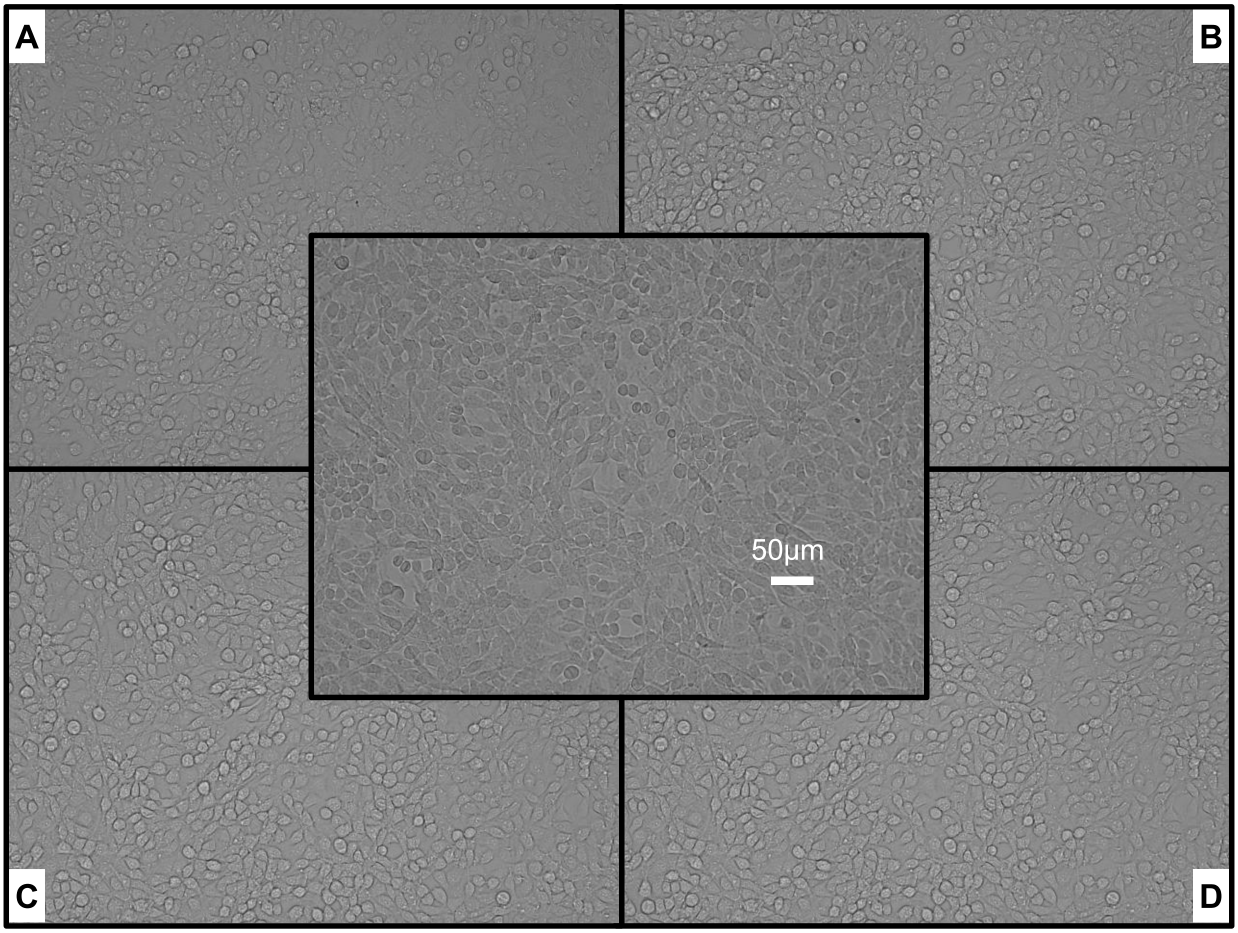

Figure S39. Confocal images of the C6 cells following 24 hours of incubation with **RuP** at (A) 0.2μM, (B) 2μM, (C) 20μM, and (D) 40μM. Central image are DMSO control cells.

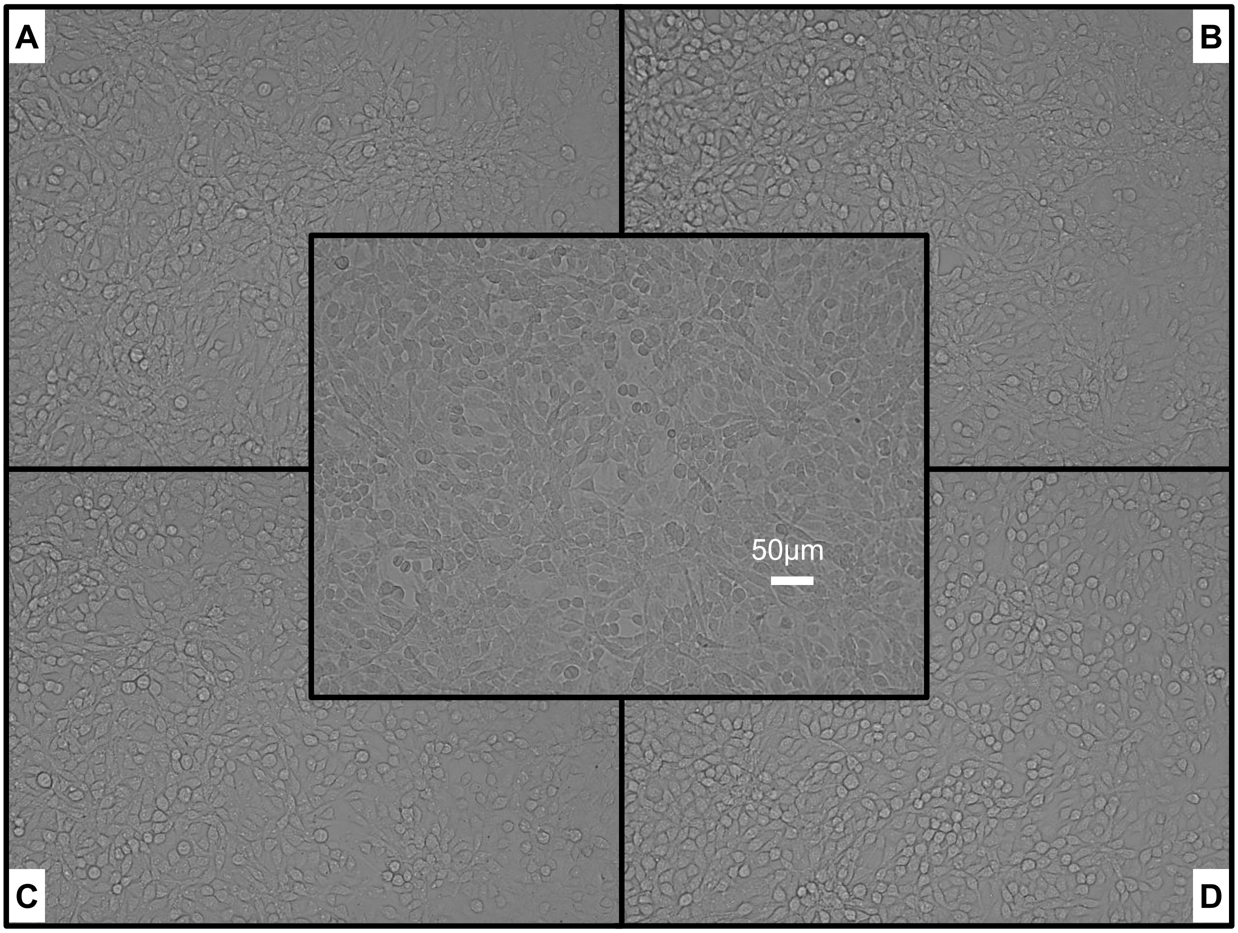

Figure S40. Confocal images of the C6 cells following 24 hours of incubation with **RuPA** at (A) 0.2µM, (B) 2µM, (C) 20µM, and (D) 40µM. Central image are DMSO control cells.

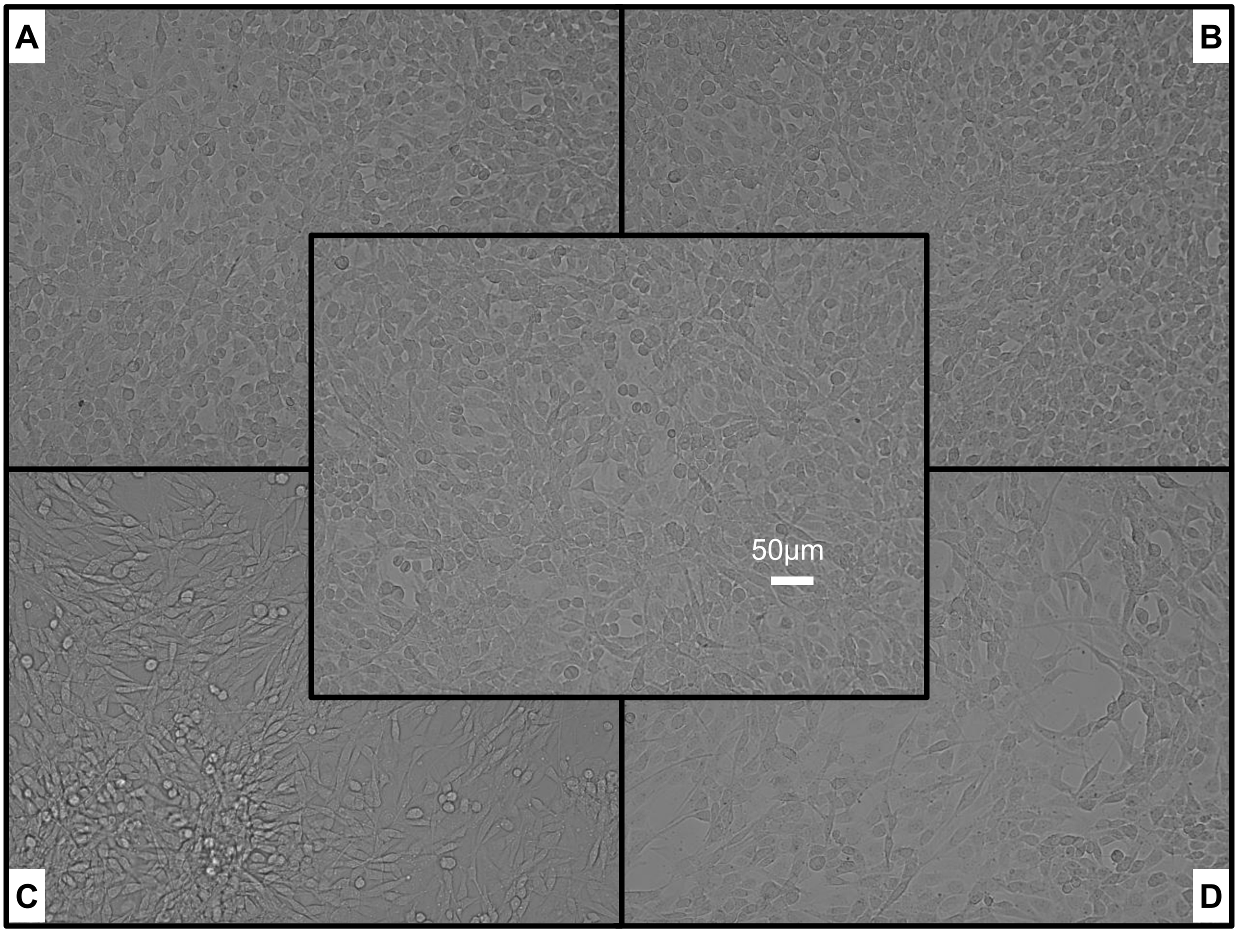

Figure S41. Confocal images of the C6 cells following 24 hours of incubation with **RuPMeO** at (A) 0.2µM, (B) 2µM, (C) 20µM, and (D) 40µM. Central image are DMSO control cells.

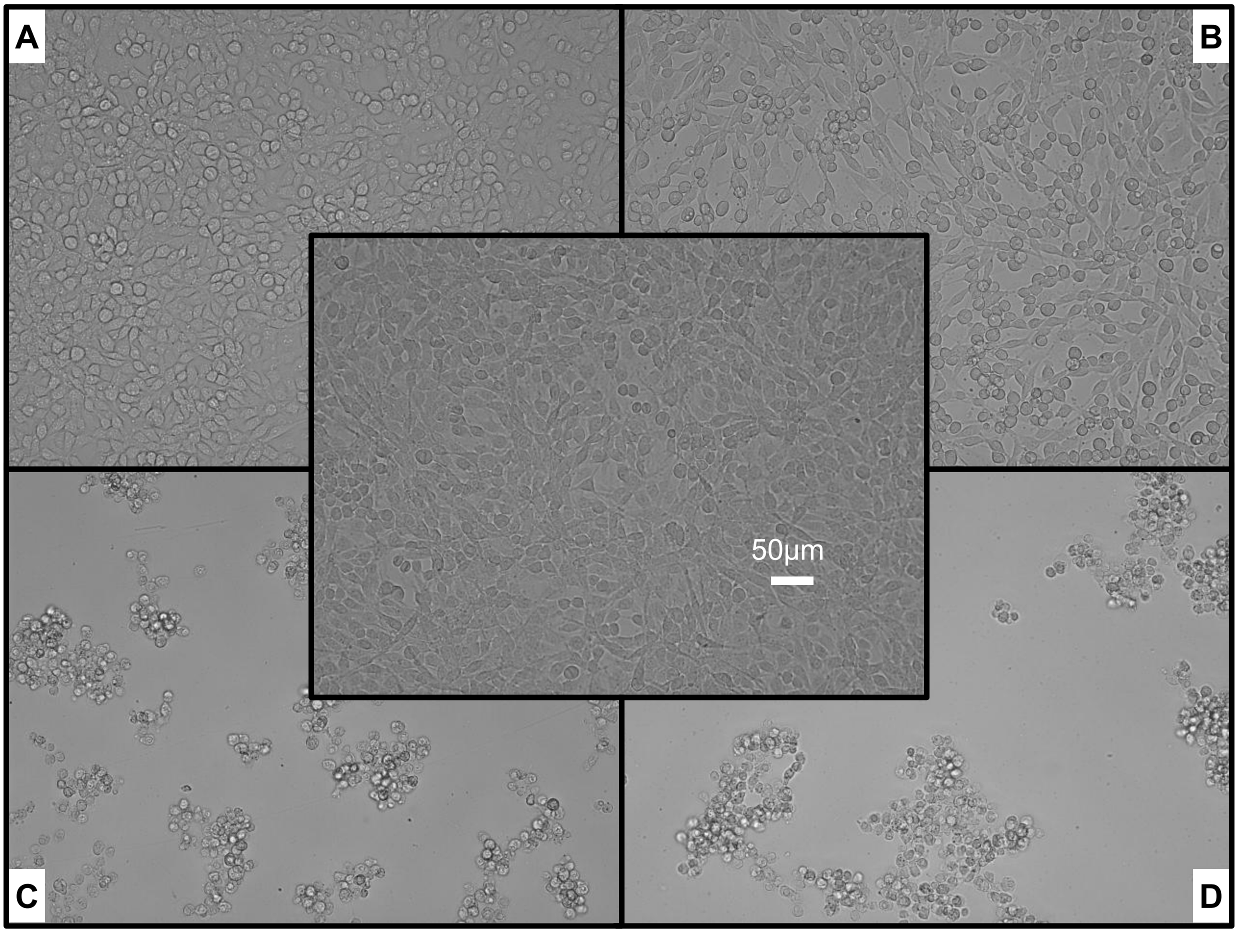

Figure S42. Confocal images of the C6 cells following 24 hours of incubation with **RuPPh** at (A) 0.2μM, (B) 2μM, (C) 20μM, and (D) 40μM. Central image are DMSO control cells.

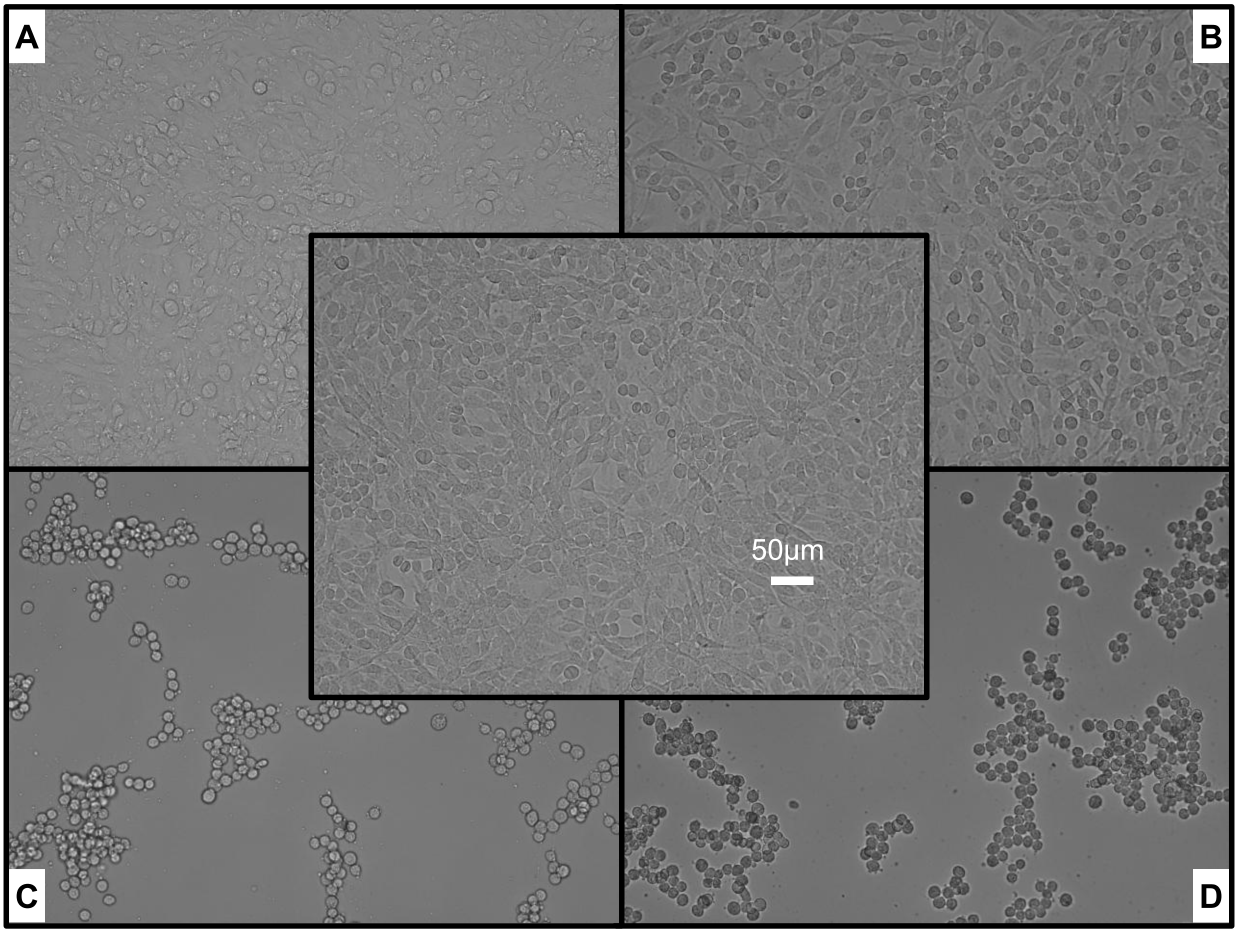

Figure S43. Confocal images of the C6 cells following 24 hours of incubation with **RuDppz** at (A) 0.2µM, (B) 2µM, (C) 20µM, and (D) 40µM. Central image are DMSO control cells.

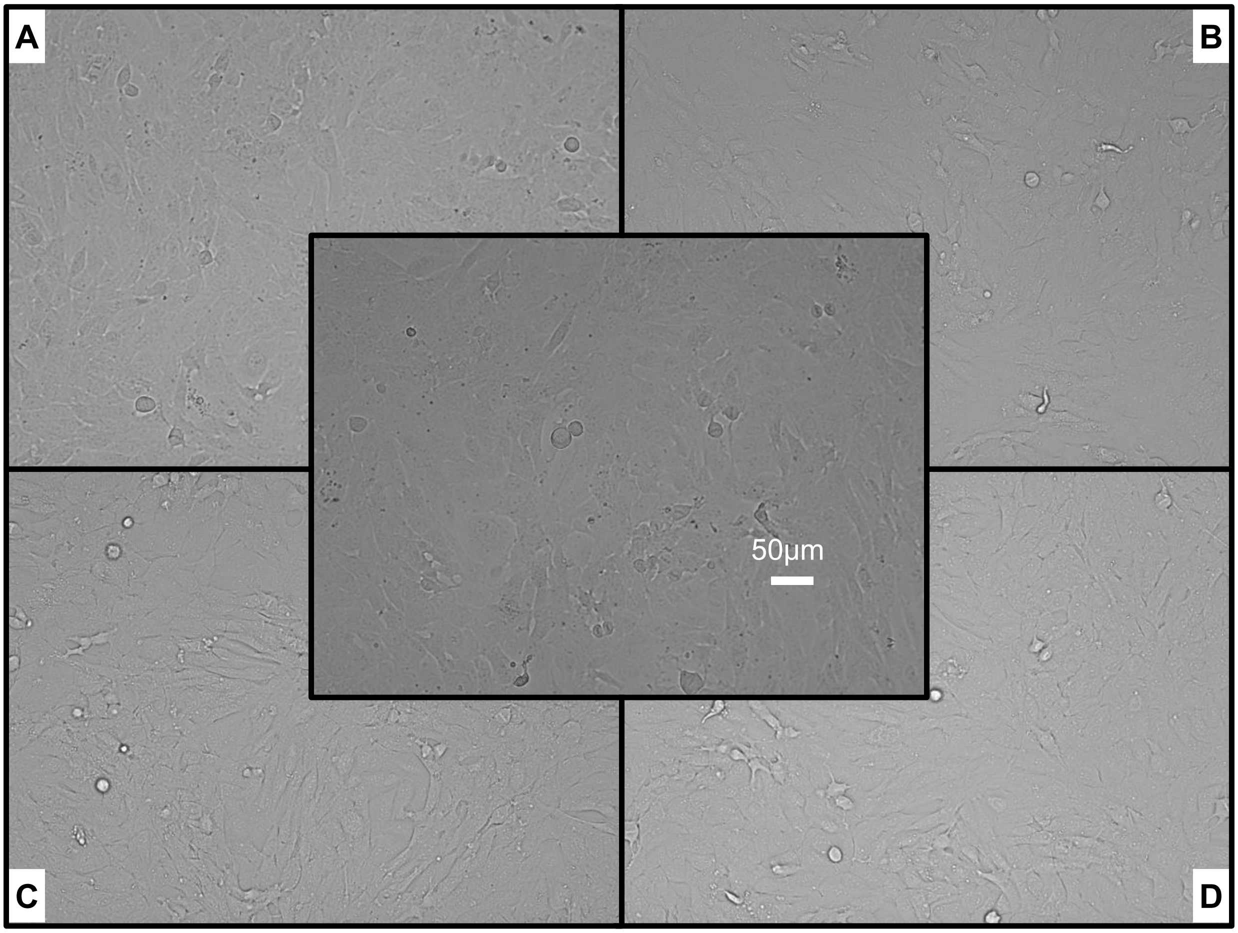

Figure S44. Confocal images of the PC12 cells following 24 hours of incubation with **RuP** at (A) 0.2µM, (B) 2µM, (C) 20µM, and (D) 40µM. Central image are DMSO control cells.

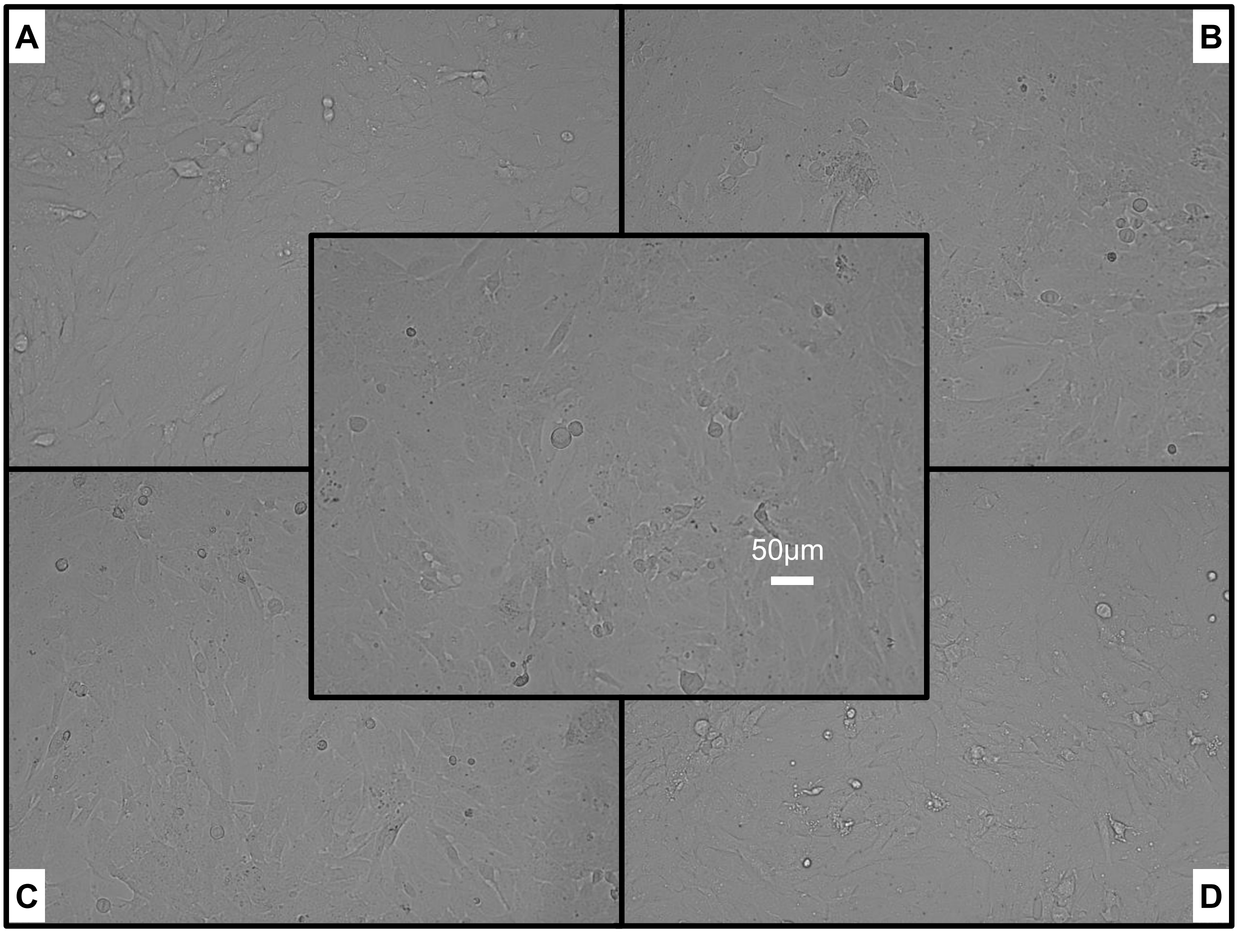

Figure S45. Confocal images of the PC12 cells following 24 hours of incubation with **RuPA** at (A) 0.2µM, (B) 2µM, (C) 20µM, and (D) 40µM. Central image are DMSO control cells.

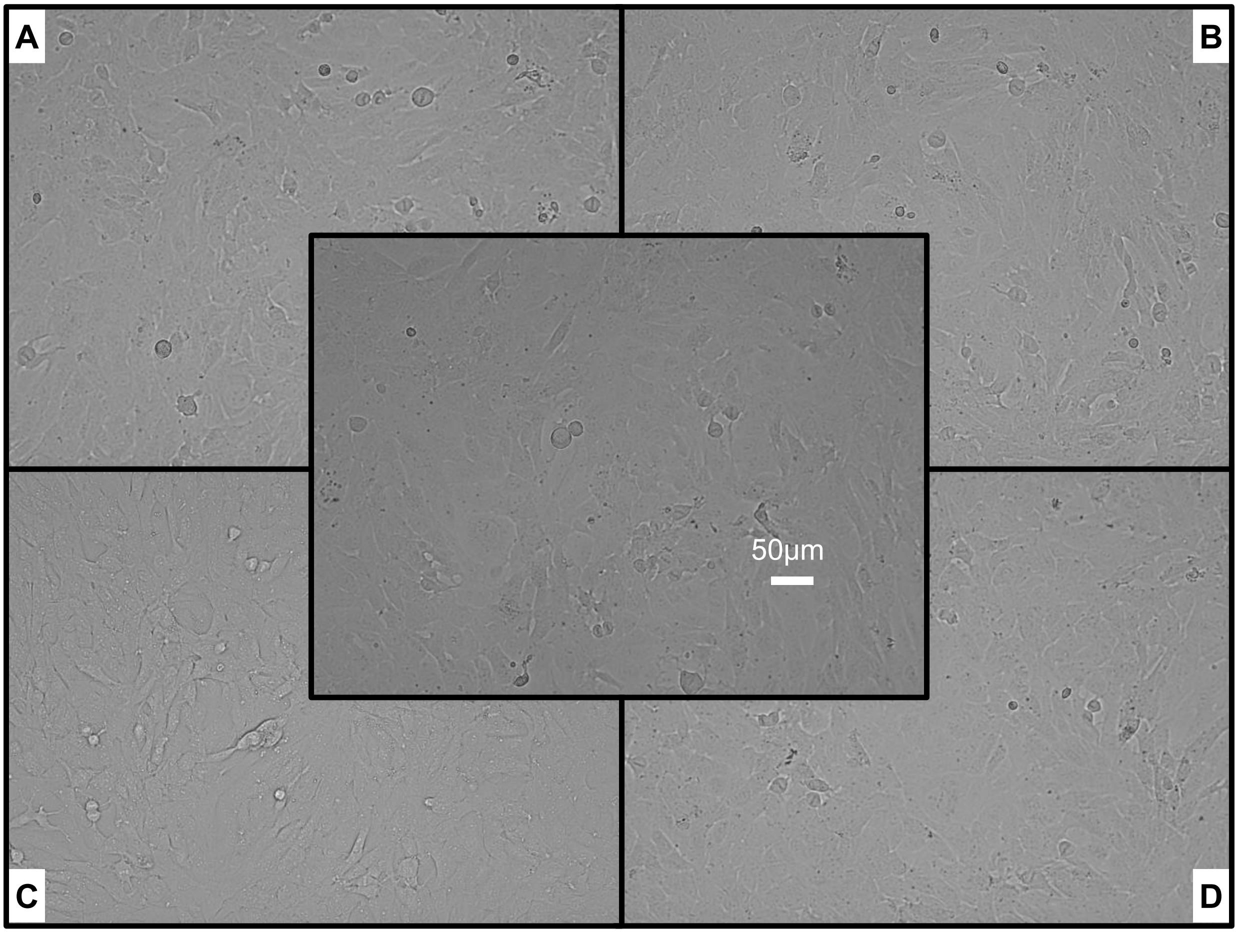

Figure S46. Confocal images of the PC12 cells following 24 hours of incubation with **RuPMeO** at (A) 0.2μM, (B) 2μM, (C) 20μM, and (D) 40μM. Central image are DMSO control cells.

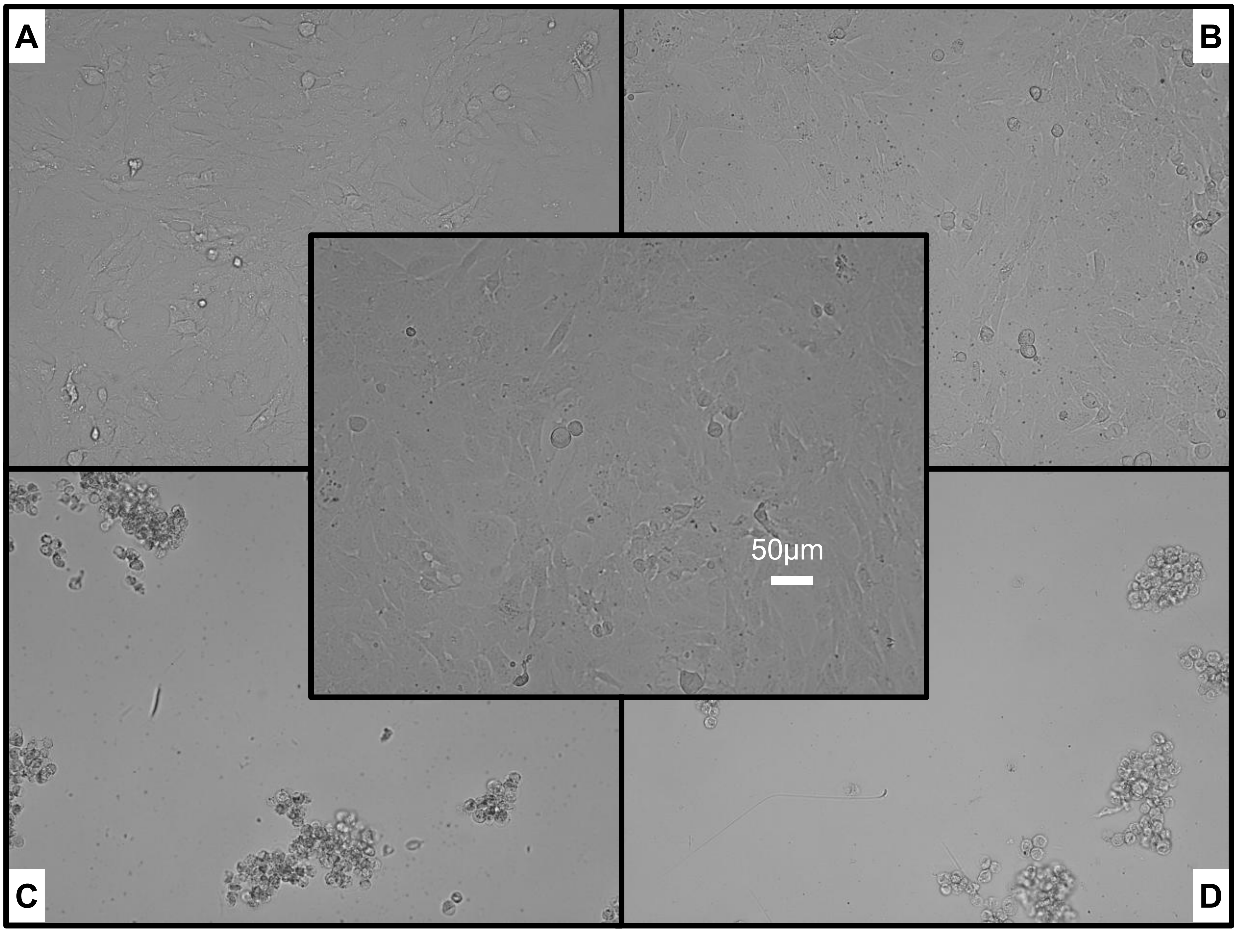

Figure S47. Confocal images of the PC12 cells following 24 hours of incubation with **RuPPh** at (A) 0.2µM, (B) 2µM, (C) 20µM, and (D) 40µM. Central image are DMSO control cells.

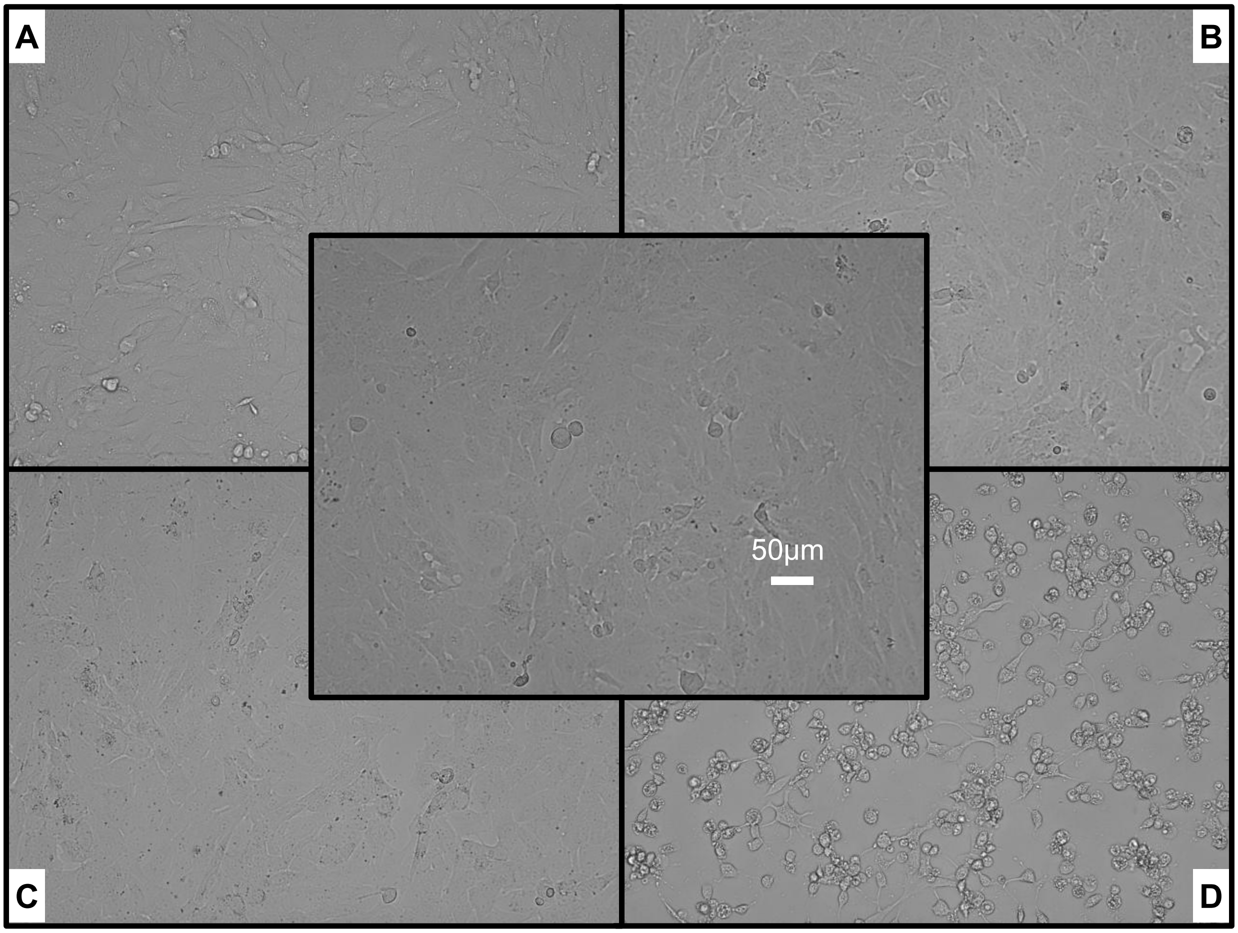

Figure S48. Confocal images of the PC12 cells following 24 hours of incubation with **RuDppz** at (A) 0.2μM, (B) 2μM, (C) 20μM, and (D) 40μM. Central image are DMSO control cells.

Table S1. Crystal data and structure refinement for **RuPA**.

CCDC Number	2489851	
Empirical formula	C22 H28.19 Cl F6 N4 O2.10 P Ru	
Formula weight	663.77	
Temperature	99.99(10) K	
Wavelength	1.54184 Å	
Crystal system	triclinic	
Space group	*P-1*	
Unit cell dimensions	*a = 7.09210(10) Å*	*a = 103.4700(10)°*
	b = 12.31010(10) Å	*b = 93.5520(10)°*
	c = 15.07760(10) Å	*g = 95.8310(10)°*
Volume	1268.51(2) $Å^3$	
Z	2	
Density (calculated)	1.738 Mg/m^3	
Absorption coefficient	7.242 mm^{-1}	
F(000)	670	
Crystal color, morphology	yellow-orange, plate	
Crystal size	0.173 x 0.109 x 0.028 mm^3	
Theta range for data collection	3.026 to 80.114°	
Index ranges	$-8 \le h \le 9$, $-15 \le k \le 14$, $-18 \le l \le 19$	
Reflections collected	42850	
Independent reflections	5429 [*R*(int) = 0.0476]	
Observed reflections	5328	
Completeness to theta = 74.504°	99.8%	
Absorption correction	Multi-scan	
Max. and min. transmission	1.00000 and 0.65087	
Refinement method	Full-matrix least-squares on F^2	
Data / restraints / parameters	5429 / 0 / 383	
Goodness-of-fit on F^2	1.044	
Final *R* indices [*I*>2sigma(*I*)]	*R*1 = 0.0289, *wR*2 = 0.0693	
R indices (all data)	*R*1 = 0.0293, *wR*2 = 0.0696	
Extinction coefficient	0.00121(9)	
Largest diff. peak and hole	0.851 and -0.673 $e.Å^{-3}$	

Table S2. Crystal data and structure refinement for **RuPMeO**.

CCDC Number	2449928	
Empirical formula	C24 H26 Cl F6 N2 O2 P Ru	
Formula weight	655.96	
Temperature	100.00(10) K	
Wavelength	1.54184 Å	
Crystal system	orthorhombic	
Space group	*Pbca*	
Unit cell dimensions	*a = 14.44500(10) Å*	*a = 90°*
	b = 14.88420(10) Å	*b = 90°*
	c = 23.39920(10) Å	*g = 90°*
Volume	5030.88(5) $Å^3$	
Z	8	
Density (calculated)	1.732 Mg/m^3	
Absorption coefficient	7.273 mm^{-1}	
F(000)	2640	
Crystal color, morphology	yellow-orange, block	
Crystal size	0.153 x 0.127 x 0.074 mm^3	
Theta range for data collection	3.778 to 80.159°	
Index ranges	$-18 \le h \le 15$, $-18 \le k \le 18$, $-29 \le l \le 26$	
Reflections collected	44968	
Independent reflections	5403 [*R*(int) = 0.0386]	
Observed reflections	5069	
Completeness to theta = 74.504°	99.9%	
Absorption correction	Multi-scan	
Max. and min. transmission	1.00000 and 0.76175	
Refinement method	Full-matrix least-squares on F^2	
Data / restraints / parameters	5403 / 0 / 340	
Goodness-of-fit on F^2	1.095	
Final *R* indices [*I*>2sigma(*I*)]	*R*1 = 0.0286, *wR*2 = 0.0778	
R indices (all data)	*R*1 = 0.0305, *wR*2 = 0.0791	
Extinction coefficient	0.000057(14)	
Largest diff. peak and hole	0.536 and -0.675 e.$Å^{-3}$	
